# Activity‐based proteomics uncovers suppressed hydrolases and a *neo*‐functionalised antibacterial enzyme at the plant–pathogen interface

**DOI:** 10.1111/nph.18857

**Published:** 2023-03-29

**Authors:** Daniela J. Sueldo, Alice Godson, Farnusch Kaschani, Daniel Krahn, Till Kessenbrock, Pierre Buscaill, Christopher J. Schofield, Markus Kaiser, Renier A. L. van der Hoorn

**Affiliations:** ^1^ The Plant Chemetics Laboratory, Department of Biology University of Oxford Oxford OX1 3RB UK; ^2^ ZMB Chemical Biology, Faculty of Biology University of Duisburg‐Essen 45117 Essen Germany; ^3^ Chemistry Research Laboratory Department of Chemistry and the Ineos Oxford Institute for Antimicrobial Research Oxford OX1 3TA UK

**Keywords:** activity‐based proteomics, apoplast, chitinase, glycosidase, hydrolases, *Nicotiana benthamiana*, PR3, *Pseudomonas syringae*

## Abstract

- The extracellular space of plant tissues contains hundreds of hydrolases that might harm colonising microbes. Successful pathogens may suppress these hydrolases to enable disease. Here, we report the dynamics of extracellular hydrolases in *Nicotiana benthamiana* upon infection with *Pseudomonas syringae*.
- Using activity‐based proteomics with a cocktail of biotinylated probes, we simultaneously monitored 171 active hydrolases, including 109 serine hydrolases (SHs), 49 glycosidases (GHs) and 13 cysteine proteases (CPs).
- The activity of 82 of these hydrolases (mostly SHs) increases during infection, while the activity of 60 hydrolases (mostly GHs and CPs) is suppressed during infection. Active β‐galactosidase‐1 (BGAL1) is amongst the suppressed hydrolases, consistent with production of the BGAL1 inhibitor by *P. syringae*. One of the other suppressed hydrolases, the pathogenesis‐related *Nb*PR3, decreases bacterial growth when transiently overexpressed. This is dependent on its active site, revealing a role for *Nb*PR3 activity in antibacterial immunity*.* Despite being annotated as a chitinase, *Nb*PR3 does not possess chitinase activity and contains an E112Q active site substitution that is essential for antibacterial activity and is present only in *Nicotiana* species.
- This study introduces a powerful approach to reveal novel components of extracellular immunity, exemplified by the discovery of the suppression of *neo*‐functionalised *Nicotiana*‐specific antibacterial *Nb*PR3.

## Introduction

Plant pathogens encounter a highly hydrolytic environment when they colonise the extracellular space (apoplast) in plant tissues. Plants secrete hundreds of hydrolytic enzymes including proteases, glucosidases and lipases, many of which accumulate to high levels during defence. These pathogenesis‐related (PR) proteins include chitinases, glucanases and proteases (van Loon *et al*., 2006; Doehlemann & Hemetsberger, 2013; Wang *et al*., 2020). In response, most pathogens secrete hydrolase inhibitors when colonising the apoplast to manipulate the host and cause disease. For instance, the oomycete plant pathogen *Phytophthora infestans* secretes Epi1 and Epi10, which are Kazal‐like protease inhibitors that suppress defence‐related subtilisin‐like protease P69B (Tian *et al*., 2004, 2005; Tian & Kamoun, 2005). *Phytophthora infestans* also secretes cystatin‐like protease inhibitors (EpiCs) that target papain‐like cysteine proteases (PLCPs) in the tomato apoplast (Tian *et al*., 2007). Tomato PLCPs are also suppressed by Cip1 and Avr2, secreted by the bacterium *Pseudomonas syringae* and the fungus *Cladosporium fulvum*, respectively (Rooney *et al*., 2005; Shabab *et al*., 2008; van Esse *et al*., 2008; Shindo *et al*., 2016). Hydrolase inhibition is not limited to proteases. Soybean pathogen *P. sojae* produces glucanase inhibitor GIP1 (Glucanase Inhibitory Protein 1; Rose *et al*., 2002) and the fungal corn pathogen *Ustilago maydis* produces Protein Essential during Penetration 1 (PEP1) to inhibit secreted maize peroxidase POX12, thereby preventing the oxidative burst associated with defence (Hemetsberger *et al*., 2012). Taken together, hydrolase inhibition in the apoplast by pathogen‐derived molecules is a common infection strategy used to manipulate the host.

We hypothesised that suppressed hydrolases play an important role in immunity. To test this, we used activity‐based protein profiling (ABPP), which allows monitoring of protein activities without previous knowledge of substrates or enzyme purification (Cravatt *et al*., 2008; Morimoto & van der Hoorn, 2016; Benns *et al*., 2021). ABPP involves the incubation of a proteome with a chemical probe, which contains a warhead that covalently reacts with the active site; a linker; and a tag to facilitate detection (Morimoto & van der Hoorn, 2016). In combination with analysis by mass spectrometry (MS), ABPP‐MS has been applied to study dynamic changes in the activities of serine hydrolases (SHs) in tomato upon infection with *C. fulvum* and *Ralstonia solanacearum*, as well as in *Arabidopsis thaliana* (Arabidopsis) upon infection with *Botrytis cinerea* (Kaschani *et al*., 2009; Sueldo *et al*., 2014; Planas‐Marquès *et al*., 2018). Here, we greatly increased the power of ABPP‐MS with a cocktail of biotinylated probes and taking advantage of a unique model pathosystem.

The interaction between *Nicotiana benthamiana* and the bacterial pathogen *Pseudomonas syringae* pv *tomato* DC3000 (*Pto*DC3000) provides an ideal system to study hydrolase suppression in the apoplast. The extraction of apoplastic fluid from *N. benthamiana* is a relatively simple procedure, ensuring high yield and purity. *Nicotiana benthamiana* is susceptible to the model plant pathogen *Pto*DC3000 when it lacks the type‐III effector hopQ1‐1 (*ΔhQ*), which would otherwise trigger immunity via the Roq1 immune receptor of *N. benthamiana* (Schultink *et al*., 2007; Wei *et al*., 2007). Using the *N. benthamiana*‐*Pto*DC3000 model pathosystem and a fluorescent probe targeting glycosidases, we previously discovered the suppression of BGAL1, an apoplast‐localised β‐galactosidase (Buscaill *et al*., 2019). BGAL1 participates in flagellin de‐glycosylation and thereby plays a key role in the release of the flagellin elicitor that is universally recognised in plants (Buscaill *et al*., 2019). The activity of BGAL1 is suppressed by a small molecule inhibitor produced by *Pto*DC3000 to promote disease (Buscaill *et al*., 2019). This discovery illustrates that suppressed hydrolases play important roles in plant immunity. Here, we explored hydrolase dynamics in apoplastic fluid of infected plants using ABPP‐MS with a cocktail of biotinylated probes to uncover additional suppressed hydrolases during infection.

## Materials and Methods

### Bacterial infection and apoplastic fluid isolation – *Pseudomonas syringae*

Van Hall pathovar tomato DC3000 lacking *hopQ1‐1* (*Pto*DC3000(*ΔhQ*) was grown in liquid LB medium (rifampicin 25 μg ml^−1^) overnight 28°C, 220 rpm). The next morning, the bacterial density was measured, and the culture was brought to OD_600_ = 0.001 with distilled, sterile (MilliQ) water. Cultures were infiltrated in fully expanded leaves of 4–5‐wk‐old *N. benthamiana* Domin using a needleless syringe. Up to two leaves were infected per plant, and MilliQ water was used for the mock treatment. Apoplastic fluid was extracted from infected leaves, 2 d after bacterial infection, as described previously (Joosten, 2012; Hong & Van der Hoorn, 2014). Briefly, leaves were harvested and submerged in MilliQ water and ice, with the abaxial side facing down. A vacuum was applied with a pump and subsequently released to allow water intake. Leaves were then rolled into a 50‐ml syringe without plunger, placed into a 50 ml tube and centrifuged at 1500 ***g*** for 25 min at 4°C, with slow acceleration and de‐acceleration of the rotor. Apoplastic fluid was recovered from the bottom of the 50 ml tube and processed immediately.

### Large‐scale labelling and affinity purification

The experiment involved four technical replicates for each biological treatment (mock, infected and no‐probe control), with each technical replicate corresponding to the apoplastic fluid isolated from 15 plants, two leaves per plant. The experiment was repeated in a subsequent year.

Four–five‐week‐old *N. benthamiana* plants were infiltrated with *Pto*DC3000(*ΔhQ*) or water (mock), and apoplastic fluid was extracted at 2 days postinoculation (dpi) as described previously. Three milliliters of the freshly obtained apoplastic fluid was labelled with the ‘probe cocktail’ (FP‐biotin, JJB111, DCG04 and DK‐D04) in a reaction mixture containing 50 mM NaAc pH 5, 5 mM DTT and 4 μM of each probe. Labelling was performed at room temperature for 4 h with constant rotation. For the no‐probe control, an equal volume of DMSO was added to a mixture of 1.5 ml of both AFs. To stop the labelling reaction, proteins were precipitated with chloroform : methanol (Wessel & Flügge, 1984) as follows: one volume of ice‐cold chloroform, three volumes of ice‐cold water and four volumes of ice‐cold methanol were added. The samples were vortexed and subsequently centrifuged (3000 ***g***, 30 min, 4°C). The precipitated proteins were resuspended in 2 ml of 1.2% SDS in 1× PBS (Life Technologies) by pipetting, and the solution was further diluted to 0.2% SDS by adding 1× PBS buffer. Proteins were then denatured by heating at 95°C for 5 min. To precipitate labelled proteins, 130 μl of avidin beads (A9207; Sigma) was added to each labelling reaction and incubated for 1 h at room temperature while rotating, after which the beads were spun down for 10 min at 400 ***g***. The supernatant was removed, and the beads were washed five times with 10 ml of 1% SDS and then twice with 10 ml of MS‐grade water. The beads were then transferred to a protein LoBind tube (Z666505‐100EA; Eppendorf, Hamburg, Germany).

### Synthesis of DK‐D04

All chemicals and solvents were from Sigma‐Aldrich, ABCR (Karlsruhe, Germany) and Fluorochem (Hadfield, UK). Fmoc‐Asp‐AOMK was synthesised according to a literature procedure (Dolle *et al*., 1994). The synthesis of DK‐D04 (I939, Biotin‐PD‐AOMK, Supporting Information Fig. S1A) was carried out on solid support, according to a general SPPS procedure, following Fmoc‐strategy. Couplings have been conducted in a syringe reactor using the corresponding Fmoc‐building blocks (3 eq.), HOBt (3 eq.), DIC (3 eq.) and a reaction time of 2 h at room temperature, while the resin suspension was agitated on an orbital shaker. Fmoc‐deprotection was achieved by the addition of a 5% (v/v) solution of diethylamine in DMF and agitation of the resulting suspension for 15 min. The general washing procedure involved alternated washing of the resin with DMF (3×), MeOH (3×) and DCM (3×). 2‐Chlorotrityl resin (1 eq.) was placed in a flame‐dried flask under an argon atmosphere. Fmoc‐Asp‐AOMK (4 eq.) dissolved in anhydrous DCM and DIPEA (5 eq.) was added, and the suspension was shaken overnight at room temperature. Methanol was added, and stirring was continued for additional 30 min. The resin was transferred into a syringe reactor and washed according to the general procedure. Resin loading was determined by Fmoc‐loading test (0.8 mmol g^−1^). For this synthesis, 250 mg (0.2 mmol) of Fmoc‐Asp‐AOMK loaded 2‐Chlorotrityl resin was utilised. A Fmoc‐deprotection and general washing was carried out and a solution of Fmoc‐6‐Ahx‐OH (212 mg, 0.6 mmol), HOBt (81 mg, 0.6 mmol), DIC (76 mg, 0.6 mmol, 93 μl) in DMF (10 ml) was utilised for the next coupling step. Fmoc‐deprotection and general washing was carried out, and coupling was continued using a solution of Biotin (147 mg, 0.6 mmol), HOBt (81 mg, 0.6 mmol), DIC (76 mg, 0.6 mmol, 93 μl) in DMF (10 ml). The crude product was cleaved off the resin utilising TFA (3 ml) and agitation at room temperature for 1 h. The obtained crude product was purified by reversed‐phase HPLC (H_2_O : ACN, 0.1% TFA; gradient: from 3% to 80%). The desired product was isolated a colourless powder. Yield: 0.73 mg (0.9 μmol). LC–MS (ESI): *m*/*z* = calcd for C_41_H_61_N_6_O_10_S^+^ [M + H]^+^ 829.42, found 829.29. HRMS (ESI): *m*/*z* = calcd for C_41_H_61_N_6_O_10_S^+^ [M + H]^+^ 829.41644, found 829.41612.

### Synthesis of TK009

Commercially available E64 Azide (Toronto Research Chemicals, Toronto, Ontario, Canada, 1 mg, 2.7 μmol, 1 eq.) was dissolved in acetonitrile (100 μl). An aliquot of an aqueous 100 mM CuI solution (16.2 μl, 1.6 μmol, 0.6 eq.) and DIPEA (1.9 μl, 10.8 μmol, 4 eq.) were added. In a second flask, commercially available BDP‐630/650‐alkyne (5 mg; Lumiprobe, Hannover, Germany) was dissolved in acetonitrile (100 μl) and an aliquot of this mixture (52.7 μl, 5.4 μmol, 2 eq.) was added to the first reaction solution. The resulting solution was stirred at room temperature for 16 h after which reaction control by LC–MS indicated completion of the reaction. The desired product was isolated from the solution by injection into a preparative HPLC equipped with a RP‐C_18_ column and run at a flow of 20 ml and with the following gradient program (all solvents contained 0.1% (v/v) TFA): 90% H_2_O/10% ACN to 30% H_2_O/70% ACN in 3 min, to 25% H_2_O/75% ACN in 20 min. Product‐containing fractions were pooled and lyophilised to yield 1.5 mg (64%) of TK009 (I912, Cy5‐E64, Fig. S1B). LC–MS (ESI): *t*_R_ = 10.09 min, *m*/*z* calculated for C_42_H_48_BF_2_N_8_O_7_S [M + H]^+^: 857.34, found 857.20.

### On‐bead trypsin digestion

The beads were resuspended in 250 μl of 8 M urea dissolved in 50 mM Tris–HCl pH 8. To reduce disulphide bridges, 12.5 μl of 200 mM DTT was added and beads were incubated at 65°C for 15 min while shaking. The beads were then cooled to 35°C. For the alkylation step, 12.5 μl of 400 mM IAA (iodoacetamide) was added and incubated at 35°C for 30 min while shaking and in the dark. Trypsin (Gold Mass spectrometry Grade; Promega, Madison, WI, USA) was reconstituted according to the manufacturers' instructions and added to the beads. Trypsin digestion was performed overnight at 37°C with shaking. After digestion, the beads were shortly spun down at low speed and the supernatant (containing the peptides) was transferred to a new protein LoBind tube. Beads were washed with 50 μl of MS‐grade water and spun down, and the supernatant was combined with the first supernatant. trifluoroacetic acid (TFA) was added to the peptides to a final concentration of 0.5–1% (v/v). Before mass spectrometry analysis, peptides were purified using Sep‐Pak C18 columns (WAT020515; Thermo Fisher, Waltham, MA, USA) following the instructions provided by the manufacturer.

### Mass spectrometry analysis

Experiments were performed on an Orbitrap Elite instrument (Thermo Fisher Scientific, Waltham, MA, USA), coupled to an EASY‐nLC 1000 liquid chromatography (LC) system (Thermo) operated in the one‐column mode. The analytical column was a fused silica capillary (75 μm × 32 or 36 cm) with an integrated PicoFrit emitter (New Objective, Littleton, MA, USA) packed in‐house with Reprosil‐Pur 120 C18‐AQ 1.9 μm resin (Dr Maisch, Ammerbuch, Germany). The analytical column was encased by a column oven (Sonation GmbH, Biberach, Germany) and attached to a nanospray flex ion source (Thermo). The column oven temperature was adjusted to 45°C during data acquisition and at 30°C in all other modes. The LC was equipped with two mobile phases: solvent A (0.1% (v/v) formic acid, FA, in water) and solvent B (0.1% FA in acetonitrile). All solvents were of UPLC grade (Sigma). Peptides were directly loaded onto the analytical column with a flow rate *c*. 0.5–0.8 μl min^−1^, which did not exceed 980 bar. Peptides were subsequently separated on the analytical column by running a 140 min gradient of solvent A and solvent B (start with 7% (v/v) B; gradient 7–35% B for 120 min; gradient 35% to 100% B for 10 min and 100% B for 10 min) at a flow rate of 300 nl min^−1^. The mass spectrometer was set in the positive ion mode and operated using Xcalibur software (v.2.2 SP1.48). Precursor ion scanning was performed in the Orbitrap analyser (FTMS; Fourier Transform Mass Spectrometry) in the scan range of *m*/*z* 300–1500 or 1800 and at a resolution of 60 000 with the internal lock mass option turned on (lock mass was 445.120025 *m*/*z*, polysiloxane; Olsen *et al*., 2005). Product ion spectra were recorded in a data‐dependent fashion in the ion trap (ITMS) in a variable scan range and at a rapid scan rate. The ionsation potential was set to 1.8 kV. Peptides were analysed using a repeating cycle consisting of a full precursor ion scan (1.0 or 3.0 × 10^6^ ions or 30 or 50 ms) followed by 15 product ion scans (1.0 × 10^4^ ions or 50 ms) where peptides are isolated based on their intensity in the full survey scan (threshold of 500 counts) for tandem mass spectrum (MS2) generation that permits peptide sequencing and identification. The collision‐induced dissociation (CID) energy was set to 35% for the generation of MS2 spectra. During MS2 data acquisition, dynamic ion exclusion was set to 120 s with a maximum list of excluded ions consisting of 500 members and a repeat count of one. Ion injection time prediction, preview mode for the FTMS, monoisotopic precursor selection and charge state screening were enabled. Only charge states higher than 1 were considered for fragmentation.

### Peptide and protein identification using MaxQuant

RAW spectra were submitted to an Andromeda (Cox *et al*., 2011) search using MaxQuant (v.1.6.10.43) using the default settings label‐free quantification (LFQ) and match between runs being activated (Cox *et al*., 2014). MS/MS spectra data were searched against the Uniprot *Pseudomonas syringae* pv *tomato* (strain ATCC BAA‐871/DC3000; UP000002515_223283.fasta; 5426 entries, downloaded 5/25/2020) reference Proteome and the *Nicotiana benthamiana* database (12864_2019_6058_MOESM10_ESM.fasta; 74 802 entries, downloaded 2/21/2020; Kourelis *et al*., 2019). To estimate the level of contamination, all searches included a contaminants database (as implemented in MaxQuant, 245 sequences) that contains known MS contaminants. Andromeda searches allowed for oxidation of methionine residues (16 Da) and acetylation of the protein N terminus (42 Da) as dynamic modifications and the static modification of cysteine (57 Da, alkylation with iodoacetamide). The digestion mode was set to ‘specific’, enzyme specificity was set to ‘Trypsin/P’ with two missed cleavages allowed, and the instrument type in Andromeda searches was set to Orbitrap and the precursor mass tolerance to ±20 ppm (first search) and ±4.5 ppm (main search). The MS/MS match tolerance was set to ±0.5 Da and the peptide spectrum match FDR and the protein FDR to 0.01 (based on target‐decoy approach and decoy mode ‘revert’). Minimum peptide length was seven amino acids.

The minimum score for unmodified peptides was set to 0. For protein quantification, modified peptides (minimum score 40) and unique and razor peptides were allowed. Further analysis and annotation of identified peptides was done in Perseus v.1.5.5.3 (Tyanova *et al*., 2016). Only protein groups with at least two identified unique peptides over all runs were considered for further analysis. For quantification, we combined related biological replicates to categorical groups and investigated only those proteins that were found in a minimum of one categorical group at least in three out of four biological replicas. Comparison of protein group quantities (relative quantification) between different MS runs is based solely on the LFQ's as calculated by MaxQuant (MaxLFQ algorithm). Briefly, label‐free protein quantification was switched on, and unique and razor peptides were considered for quantification with a minimum ratio count of 2. Retention times were recalibrated based on the built‐in nonlinear time rescaling algorithm. MS/MS identifications were transferred between LC–MS/MS runs with the ‘Match between runs’ option in which the maximal match time window was set to 0.7 min and the alignment time window set to 20 min. The quantification is based on the ‘value at maximum’ of the extracted ion current. At least two quantitation events were required for a quantifiable protein.

### MS data analysis

Data were analysed with Perseus (Tyanova *et al*., 2016), and only proteins corresponding to *Nicotiana benthamiana* were included in the analysis. Proteins were only considered for analysis if they were identified in three of the four replicates for at least one treatment (i.e. mock or infected). Proteins enriched compared with the no‐probe control (NPC) were considered as labelled and further analysed. To identify differentially active hydrolases upon infection, we performed a *t*‐test with Benjamini–Hochberg correction for multiple testing (α = 0.05) comparing mock and infected samples. Final hydrolase list was manually curated using Pfam (El‐Gebal *et al*., 2019) to confirm correct annotation as probe target.

### Cloning of hydrolases

Overexpression constructs for six candidate hydrolases were built by Golden Gate Assembly (Engler *et al*., 2014). The full‐length genes were amplified from cDNA using primers summarised in Table S1. Binary vectors were generated in a Golden Gate reaction with *35S* promoter (pICH51288) and *35S* terminator (pICH41414) into binary backbone pJK001c (Paulus *et al*., 2020) using Bsa1 restriction sites, resulting in binary clones summarised in Table S2. Binary vectors were transformed into *Agrobacterium tumefaciens* GV3101 (pMP90) by freeze‐thawing and selection for kanamycin and gentamicin resistance. The E92A and Q112E mutants of *Nb*PR3 were generated by site‐directed mutagenesis using the primers listed in Table S1, using plasmid pAG001 as a template. The PCR product was digested with DpnI to remove template plasmid, and the remaining mutated product was transformed into *E. coli*. Sequence‐confirmed positive clones were selected and transformed into *A. tumefaciens*.

### Agroinfiltation

*Agrobacterium* cultures were grown in LB media (kanamycin 50 μg ml^−1^ and gentamicin 10 μg ml^−1^) overnight at 28°C. The next day, cultures were spun at 4000 ***g*** for 10 min at room temperature and resuspended in infiltration buffer (10 mM MgCl_2_, 10 mM MES, 150 μM acetosyringone) to a final OD_600_ 0.5. Cultures carrying *Nb*PR3 were co‐infiltrated with cultures carrying silencing suppressor P19 (Van der Hoorn *et al*., 2003), and P19 combined with empty vector (EV) was used as negative control. *N. benthamiana* plants were infiltrated at 4–5 wk old, and apoplastic fluid was extracted at 4 dpi as indicated previously (Joosten, 2012; Hong & Van der Hoorn, 2014).

### Protein analysis

Apoplastic fluid was isolated from *N. benthamiana* plants transiently overexpressing hydrolases at 4 dpi. The presence of active serine hydrolases and cysteine proteases was measured by ABPP using the FP‐TAMRA probe (Thermo Fisher Scientific) and TK009 (see above), respectively. To label hydrolases, apoplastic fluid samples were incubated in a 50 μl reaction with 0.2 μM FP‐TAMRA (for serine hydrolases), or a 250 μl reaction with 2 μM I912 (for cysteine proteases), in the presence of 5 mM DTT and 50 mM sodium acetate pH 5 at room temperature for 1 h (serine hydrolases) or 4 h (for cysteine proteases). After labelling, the reaction was stopped by precipitation in 4× ice‐cold acetone followed by resuspension in 4× gel loading buffer and heating at 90°C for 5 min. Samples were separated by SDS‐PAGE and visualised using a Typhoon scanner at Cy3 filter (serine hydrolases) or Cy5 filter (cysteine proteases). Coomassie staining was used to check protein loading. *Nb*PR3 was detected by western blot using anti‐PR3 antibody designed for tobacco PR3 isoforms (1 : 2500 in PBS‐T; Agrisera, Vännäs, Sweden) and an‐rabbit‐HRP secondary antibody (GE Healthcare, Chicago, IL, USA).

### Agromonas infection assay

*Agrobacterium* cultures were grown in LB media (kanamycin 50 μg ml^−1^ and gentamicin 10 μg ml^−1^) overnight at 28°C. The next day, cultures were spun at 4000 ***g*** for 10 min at room temperature and resuspended in infiltration buffer (10 mM MgCl_2_, 10 mM MES, 150 μM acetosyringone) to a final OD_600_ 0.5. All constructs were co‐infiltrated with the silencing suppressor P19 (Van der Hoorn *et al*., 2003), and P19 with empty vector was used as mock control. *Nicotiana benthamiana* plants were infiltrated when 3–4 wk old, and 2–3 fully expanded leaves were infiltrated per plant. Six plants were used per overexpression construct. Plants were covered overnight with a transparent lid to increase humidity. Two days later, agroinfiltrated leaves were infiltrated with 10^6^ CFU ml^−1^ *Pto*DC3000(*ΔhQ*) as indicated previously. Three days later, leaf discs were punched with a cork borer from each infected leaf and surface‐sterilised with 15% hydrogen peroxide for 2 min. Leaf discs were then washed twice in MilliQ and dried. Leaf discs were placed into a 1.5 ml safe‐lock Eppendorf tube with three 3 mm diameter metal beads and 1 ml of MilliQ. Tubes were placed in tissue lyser for 5 min at 30 Hertz s^−1^. 200 μl of the lysed tissue was transferred to the first row (A) of a 96‐well plate, and then serial 10‐fold dilutions were made until the last row (20 μl tissue +180 μl MilliQ water). Twenty microliters of undiluted tissue and serial dilutions was plated on LB‐agar plates containing Pseudomonas CFC Agar Supplement (10 μg ml^−1^ cetrimide, 10 μg ml^−1^ fucidin and 50 μg ml^−1^ cephaloridine, SR0103; Thermo Fisher Scientific). Plates were allowed to dry and incubated at 28°C for 2 d, and then colonies were counted.

### Endochitinase activity

Apoplastic fluid was used as an enzyme source to test endochitinase activity as described before (Hollis *et al*., 1997; Libantová *et al*., 2009). Briefly, 20 μl of apoplastic fluid was combined with 30 μl of substrate solution (4‐methylumbelliferyl‐β‐d‐*N*,*N*′,*N′′*‐triacetylchitotrioside; Sigma) to a final substrate concentration of 18 μM. Reactions were performed in a black 96‐well plate and incubated for 1 h at 37°C in the plate reader. Measurements were taken every minute for 1 h using 355 nm excitation and 450 nm emission. The positive control contained commercial chitinase from *Trichoderma viride* at a final concentration of 0.04 mg ml^−1^ (C8241; Sigma).

### Lysozyme assay

Apoplastic fluid isolated from leaves transiently expressing *Nb*PR3 was used to test lysozyme activity following the instructions of the lysozyme manufacturer. Briefly, 10 μl apoplastic fluid was mixed with 250 μl *Micrococcus lysodeikticus* cells resuspended in potassium phosphate buffer at 0.15 mg ml^−1^. Absorbance at 450 nm was recorded in Tecan plate reader at 15‐s intervals for 5 min at 25°C. The maximum linear rate of A_450_ decrease was calculated and converted into enzymatic units per μg protein. The positive control contained 0.1 μg commercial lysozyme from chicken egg white (L6876; Sigma).

## Results

### The apoplast is rich in hydrolases

**Fig. 1:**
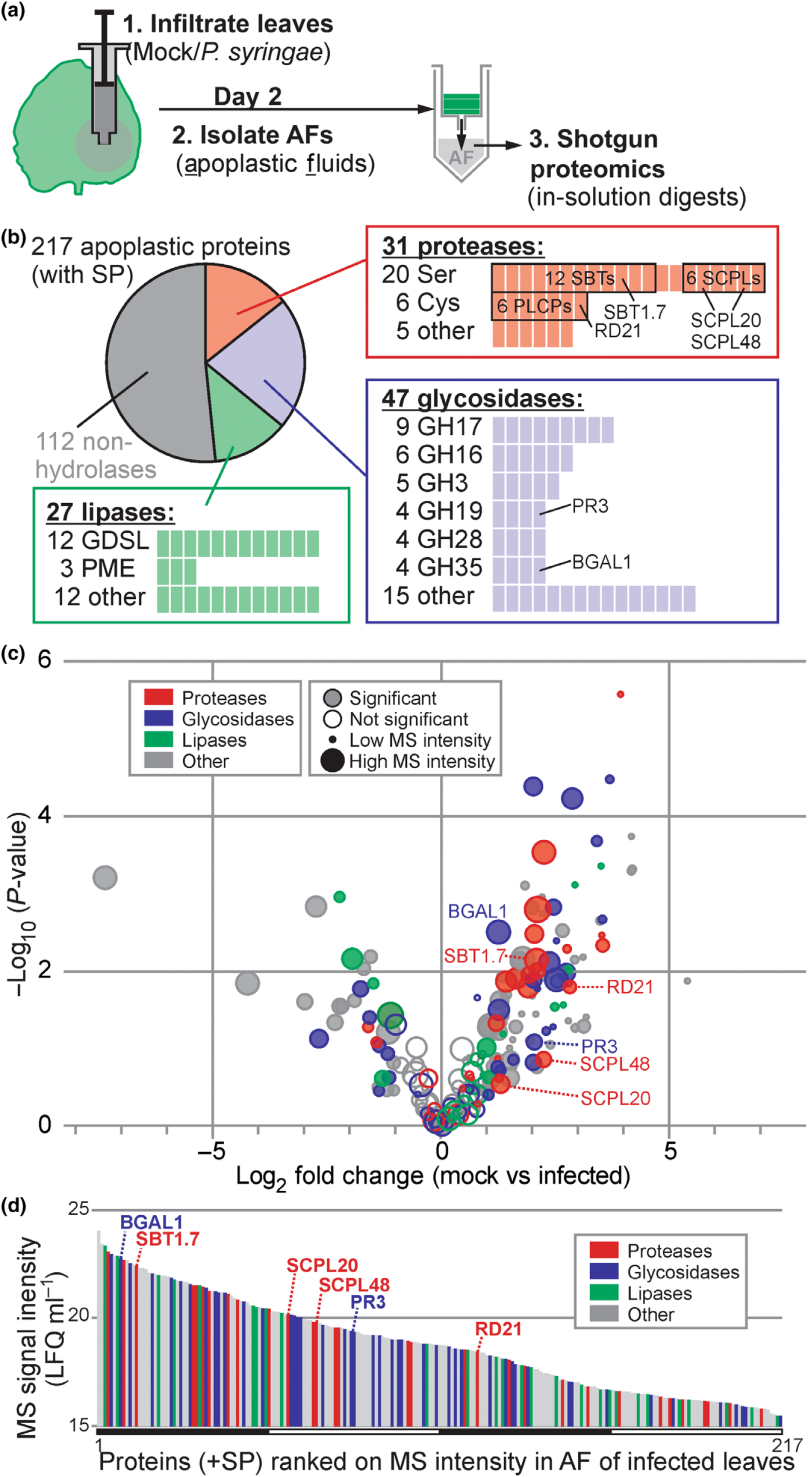
Many plant hydrolases accumulate in the apoplast following infection. (a) Experimental design. *Nicotiana benthamiana* leaves were inoculated with *Pto*DC3000(*ΔhQ*) (infected) or water (Mock) and apoplastic fluid (AF) was collected at 2‐d postinoculation (2 dpi). Proteins were digested with trypsin and analysed by mass spectrometry. (b) Many detected secreted proteins are hydrolases. Detected proteins that have a signal peptide predicted by SignalP were annotated with Pfam and classified into proteases (red), glycosidases (blue) and lipases (green) and subdivided into protein families. (c) More hydrolytic enzymes accumulate in apoplast upon infection. The *P*‐values of the *t*‐test were plotted against the fold change. The circle diameter reflects the sum of the mass spectrometry (MS) intensities, and filled circles identify proteins with a significant differential accumulation. (d) Hydrolytic enzymes are relatively abundant in the apoplast. All 217 signal peptide (SP)‐containing proteins detected by MS were ranked on average protein intensities detected in AF from mock and infected samples. The different hydrolase classes highlighted in colours, and six hydrolases are highlighted. (a–d) AF isolated from mock‐ and *Pto*DC3000(*ΔhQ*)‐infected plants were isolated from four independent experiments and analysed by mass spectrometry. label‐free quantitation (LFQ) intensities were corrected for protein concentrations measured in the respective AF samples to calculate LFQ ml^−1^. Plant proteins having a predicted SP and detected in all eight samples were retained and used for graphs (b–d). See Supporting Information Table S3 for all detected proteins from this ACE_0276 experiment.

To investigate the accumulation of hydrolases in the apoplast of infected and noninfected plants, *N. benthamiana* was infiltrated with water (mock) and *Pto*DC3000(*ΔhQ*). We extracted apoplastic fluids (AFs) at 2‐d postinfection (2 dpi), then digested the proteomes with trypsin and analysed them by LC–MS/MS. We robustly identified 217 proteins carrying a predicted signal peptide (SP, by SignalP, Almagro Armenteros *et al*., 2019). Classification of these 217 proteins using Pfam (El‐Gebal *et al*., 2019) revealed that 48% of these apoplastic proteins are hydrolases (105 proteins, Fig. 1a; Table S3). The other 112 apoplastic proteins are diverse and include eight inhibitors and 13 peroxidases.

The 105 detected hydrolases include 31 proteases, 47 glycosidases and 27 lipases (Fig. 1b). Detected apoplastic proteases belonged to the mechanistic class of Ser proteases (SPs, 20 proteins), Cys proteases (CPs, six proteins) and other proteases (five proteins; Fig. 1b). The 20 Ser proteases include 12 subtilisin‐like proteases (SBTs, S8 family) and six Ser carboxypeptidase‐like proteases (SCPLs, S10). The six Cys proteases are all papain‐like Cys proteases (PLCPs, C01). The 47 glycosidases belong to 18 different glycosyl hydrolase (GH) families, which include nine GH17 glycosidases, six GH16 glycosidases, five GH3 glycosidases and four glycosidases each from families GH19, GH28 and GH35. Finally, the 27 lipases include 12 GDSL‐lipases, three pectinacetylesterases (PAEs) and 12 other lipases. This proteome composition is similar to that of a previously reported proteome (Buscaill *et al*., 2019, ACE_0058).

Protein concentrations in the apoplast can increase 10‐fold upon infection (Table S4), so we corrected our proteomics dataset for this by calculating the MS intensity per ml of apoplastic fluid to facilitate the comparative analysis of protein concentrations. When plotted in a volcano plot, the protein concentrations of abundant hydrolases significantly increase upon infection (Fig. 1c), consistent with the well‐known accumulation of PR proteins, such as chitinases and glucanases (van Loon *et al*., 2006). Consequently, apoplastic hydrolases are a major component of the apoplastic proteome of infected plants. When ranked by MS intensity, which is an approximation for protein abundance (Cox *et al*., 2014), 30 hydrolases are amongst the top quartile of most abundant proteins in the apoplast of infected plants (Fig. 1d). In conclusion, a large proportion of the extracellular proteins encountered by pathogens during infection are hydrolytic enzymes.

### Large‐scale activity profiling of the apoplast upon bacterial infection

**Fig. 2:**
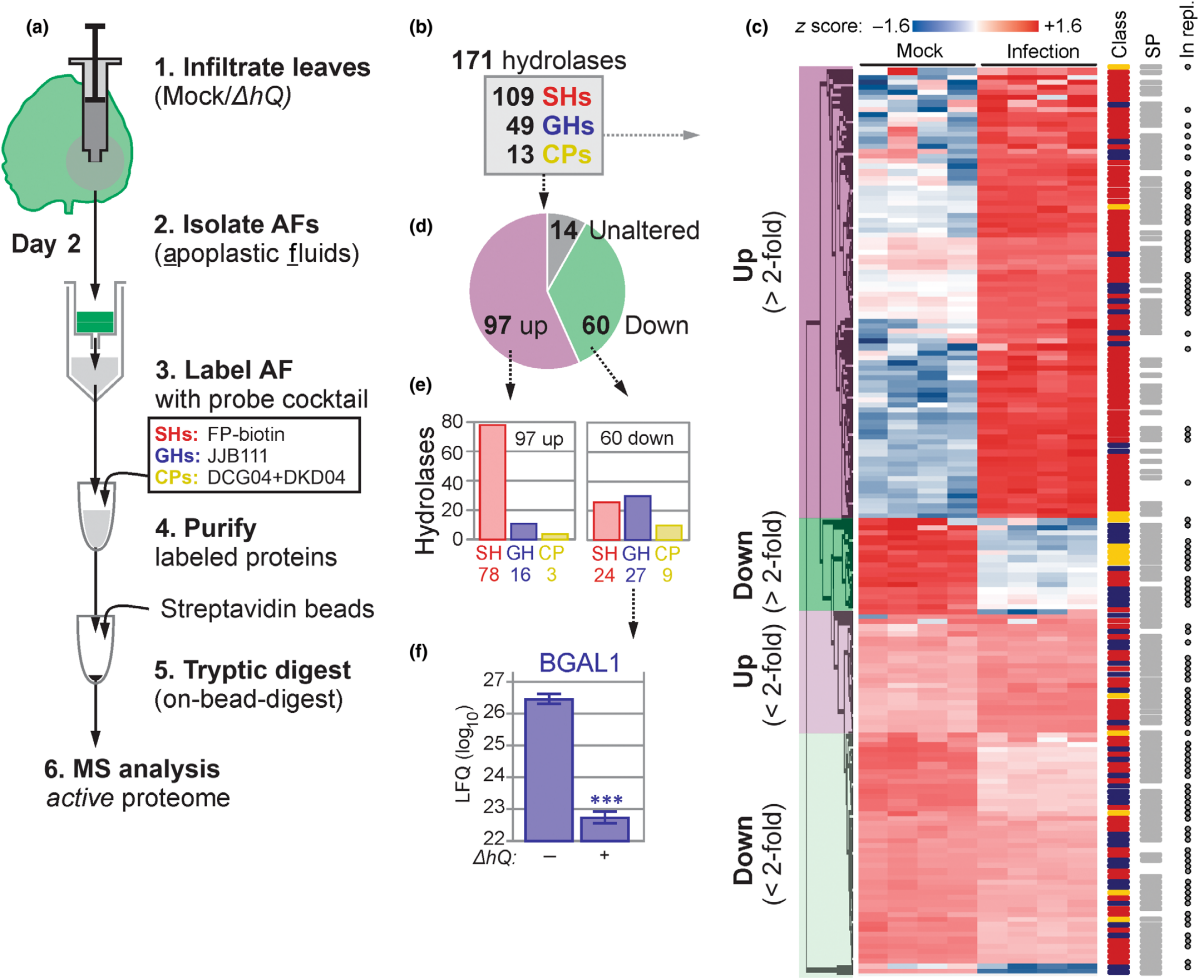
Activity‐based proteomics displays hydrolase activity dynamics upon infection. (a) Experimental procedure. *Nicotiana benthamiana* plants were infiltrated with *Pto*DC3000(*ΔhQ*) or water (Mock) and apoplastic fluid (AF) was collected at 2‐d postinfection (2 dpi). AFs were labelled with a cocktail activity‐based probes targeting active serine hydrolases (SHs, red), glycosyl hydrolases (GHs, blue) and cysteine proteases (CPs, yellow). Labelled proteins were purified and identified by mass spectrometry (MS) for *n* = 4 replicates. (b) Classification of 171 robustly detected labelled hydrolases. (c) Heatmap of the 171 detected active hydrolases detected by activity‐based proteomics (experiment ACE136), grouped by category (left) and annotated for being SH/GH/CP; having a SignalP‐predicted signal peptide (SP); and for being detected in an independent replicate MS experiment (ACE236) (right). (d) Summary of the detected differential active hydrolases showing statistically significant up or downregulation upon bacterial infection. (e) Summary of the numbers of active hydrolases belonging to each class that are up or downregulated. (f) β‐galactosidase‐1 (BGAL1) labelling is significantly downregulated upon infection. The label‐free quantitation intensities for this positive control were extracted from this dataset (ACE_0236). Error bars represent standard error from *n* = 4 biological replicates. ***, *P* < 0.001 (Students' *t*‐test).

To investigate changes in hydrolase activities in the apoplast caused by bacteria, we investigated changes in active hydrolases upon bacterial infection with ABPP‐MS. To display the active proteome, we labelled the apoplastic proteome with a cocktail of biotinylated activity‐based probes, including FP‐biotin (Liu *et al*., 1999), JJB111 (Chandrasekar *et al*., 2014) and DCG04 (Greenbaum *et al*., 2000) to display the active serine hydrolases (SHs), glycosyl hydrolases (GHs) and cysteine proteases (CPs), respectively. We also included the custom‐made Biotin‐PD‐AOMK (DK‐D04, Fig. S1A) to detect vacuolar processing enzymes (VPEs), which have been detected in apoplastic fluids (Sueldo *et al*., 2014). Biotinylated proteins were purified and analysed by MS in *n* = 4 replicates (Fig. 2a).

The annotation of ABPP‐MS spectra to the proteome of *N. benthamiana* (Kourelis *et al*., 2019) enabled the detection of 171 predicted probe targets that were all enriched (α = < 0.05) compared with the no‐probe control (Table S5). These probe targets included 109 SHs, 49 GHs and 13 CPs (Fig. 2b). Overall, 157 target proteins were differentially labelled between mock and *Pto*DC3000(*ΔhQ*) treatments (α = 0.05), indicating that 92% of the detected active proteome changes significantly during bacterial infection (Fig. 2c,d). Of the 97 activities that increased upon infection, 78 were SHs, 16 were GHs, and three CPs (Fig. 2e). Furthermore, we detected 60 reduced hydrolytic activities, including 24 SHs, 27 GHs and nine CPs (Fig. 2e). Overall, bacterial infection induces mostly active SHs and reduces mostly active GHs and CPs. Importantly, the suppressed hydrolases include BGAL1 (3.67‐fold downregulated, *P*‐value = 1.65E‐07), as described previously (Fig. 2f, Buscaill *et al*., 2019). These results indicate that the active apoplastic proteome undergoes large changes during bacterial infection, including 57% of the hydrolases having increased activity and 35% having reduced activity. Of the 97 increased active hydrolases, 74 had a fold change of at least 2 (FC ≥ 2), consisting of 63 SHs, nine GHs and two CPs. Likewise, 25 of the 60 reduced active hydrolases also had FC ≥ 2, consisting of six SH, 14 GHs and five CPs.

Although the general trend is that active SHs are induced and active GH and CPs are reduced, we observed many differences within each hydrolase subfamily (Fig. 2e). We identified 28 active GDSL‐like lipases, 23 (most) of which showed increased activity in infected tissues. We also identified 21 subtilisin‐like serine proteases (S8, SBTs), of which nine showed decreased activity and nine increased activity upon bacterial infection. Furthermore, we identified 18 serine carboxypeptidase‐like proteases (S10, SCPLs) of which 12 had increased activity during infection. We also detected 10 carboxylesterases (CXEs) and six pectin acetylesterases (PAEs), all of which were more active upon infection. Of the 17 detected active GH3s, seven were less active in infected tissue. We also detected six active GH79s and six GH35s, including BGAL1 (NbD029635; Buscaill *et al*., 2019). Eight of the 13 detected papain‐like proteases (C01) had a reduced activity upon infection. Both detected VPEs are less active in infected tissue. Altogether, these data demonstrate that the active apoplastic proteome changes drastically upon bacterial infection.

### One of the tested suppressed hydrolases inhibits bacterial growth

**Fig. 3:**
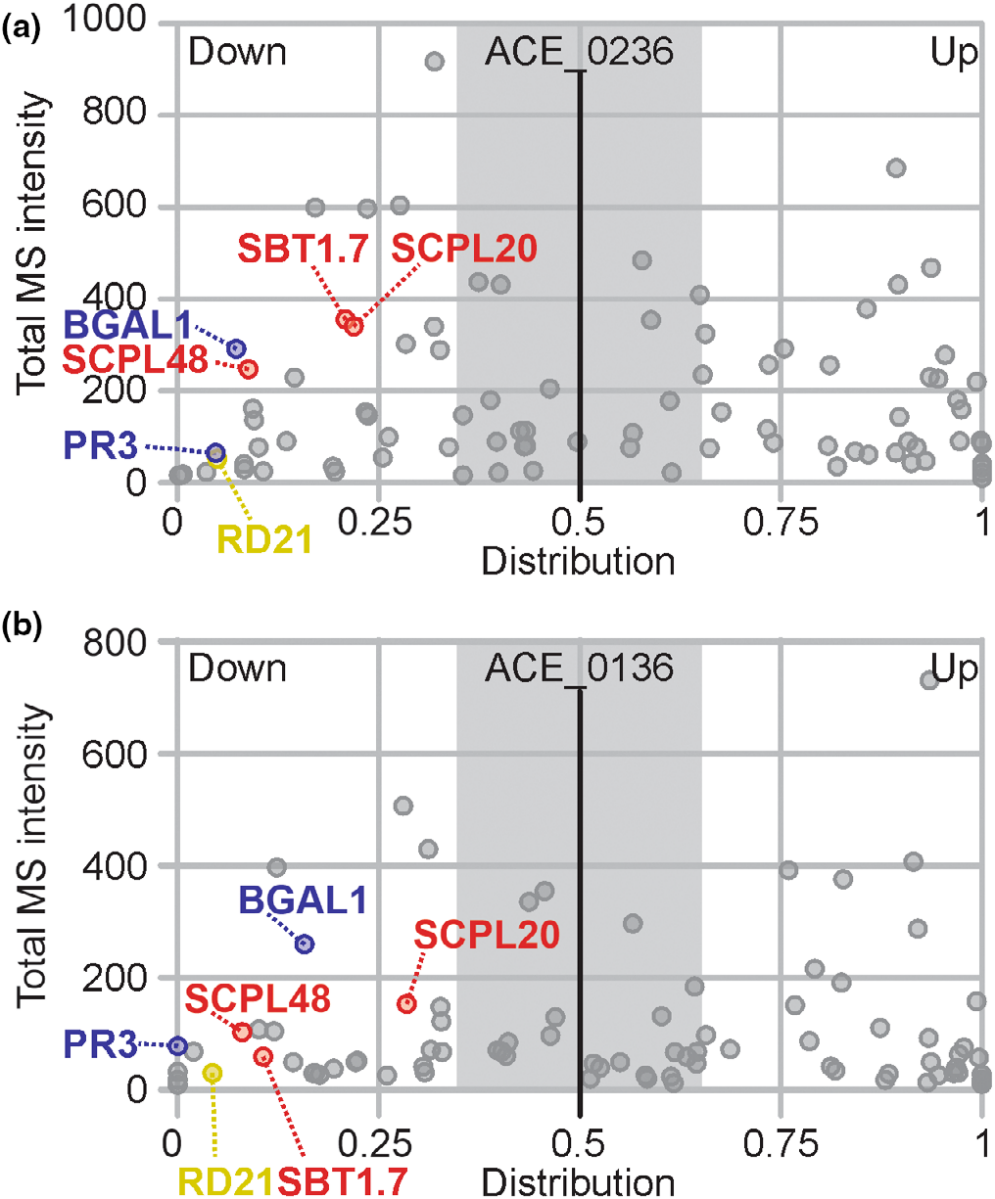
Several secreted hydrolases are consistently suppressed during infection. Distribution graphs of the active proteome from two independent experiments. Data derived from Fig. 2 (experiment ACE_0236) (a) were compared with a replicate experiment (ACE_0136, Supporting Information Fig. S3) (b) and used to select consistently suppressed hydrolases. Total mass spectrometry (MS)/MS spectra for mock and infected samples were combined and plotted against the distribution of each protein in mock and infected samples. The distribution was calculated as label‐free quantitation (LFQ)_infected_/(LFQ_mock_ + LFQ_infected_). Proteins are represented as dots, and only proteins showing the same behaviour in both biological replicates are shown. Six hydrolases (*Nb*SBT1.7a, *Nb*SCPL20, *Nb*SCPL48, *Nb*PR3, *Nb*RD21 and *Nb*BGAL1) are indicated and coloured red, blue and yellow according to hydrolase class (SH, GH and CP, respectively).

**Fig. 4:**
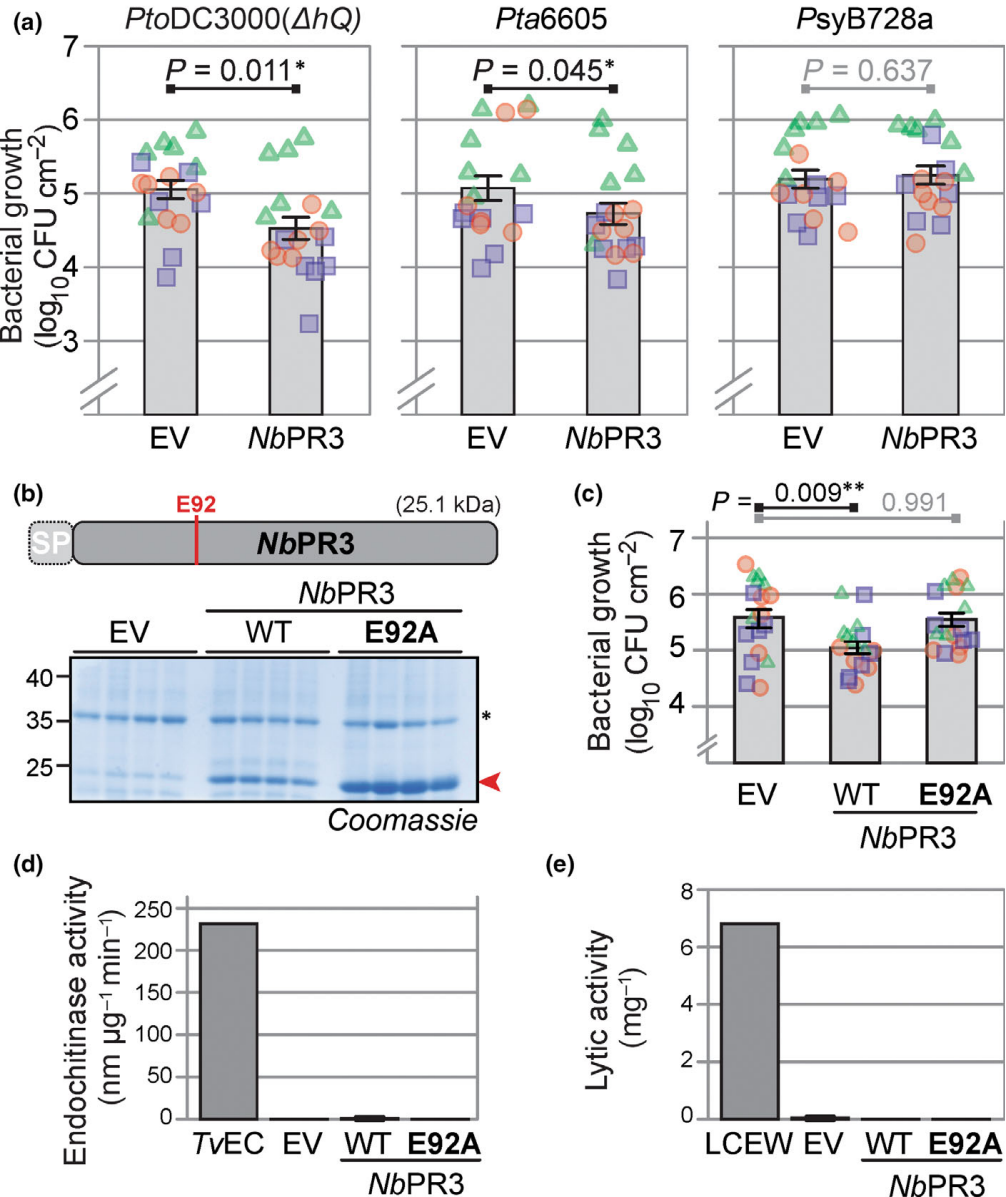
*Nb*PR3 requires its active site to reduce bacterial growth but lacks chitinase and lysozyme activity. (a) Transient *Nb*PR3 expression decreases susceptibility to *Pseudomonas syringae* pathovars *tomato* DC3000(ΔhQ) and *tabaci* 6605, but not *syringae* B728a. *Nb*PR3 and the empty vector (EV) control were transiently expressed in *Nicotiana benthamiana* by agroinfiltration. Two days later, the same leaves were infiltrated with 10^6^ CFU ml^−1^ bacteria and bacterial population densities in log_10_CFU cm^−2^ were determined after 3 d. Bars show the mean value of 18 biological replicates performed over three separate experiments, and error intervals represent the SE. *P*‐values were calculated by two‐way ANOVA followed by *post hoc* comparison using the Dunnett test to examine the effect of *Nb*PR3 overexpression on bacterial growth. (b) The *Nb*PR3(E92) mutant accumulates in the apoplast of agroinfiltrated leaves. *Nb*PR3 and its E92A mutant and the empty vector (EV) control were agroinfiltrated and apoplastic fluid (AF) was isolated at Day 4 from four different replicates, separated on protein gel and analysed by Coomassie staining. The red arrowhead indicates *Nb*PR3 protein. *, Endogenous PR2 protein, induced by agroinfiltration. (c) Transient expression of *Nb*PR3 but not its catalytic mutant suppresses bacterial growth of *Pto*DC3000(*ΔhQ*). *Nb*PR3, its catalytic site E92A mutant, and the empty vector (EV) control were transiently expressed by agroinfiltration. Two days later, the same leaf was infiltrated with 10^6^ CFU ml^−1^ *Pto*DC3000(*ΔhQ*) and bacterial population densities in log_10_CFU cm^−2^ were determined after 3 d. Bars show the mean value of 18 biological replicates performed over three separate experiments, and error intervals represent the SE. *P*‐values were calculated by two‐way ANOVA followed by *post hoc* comparison using the Dunnett test to examine the effect of *Nb*PR3 overexpression on bacterial growth. **, *P* < 0.01. (d) *Nb*PR3 lacks endochitinase activity. AF from plants transiently expressing *Nb*PR3 or its E92A mutant were incubated with 4‐MU‐GlcNac3, and the rate of hydrolysis was measured at 355ex/450em and calculated per microgram *Nb*PR3 protein estimated by Coomassie staining. The endochitinase of *Trichoderma viride* (*Tv*EC) was included as a positive control. (e) *Nb*PR3 lacks lysozyme activity. AF from plants transiently expressing *Nb*PR3 or its E92A mutant were incubated with *Micrococcus lysodeikticus* cells. The change in A_450_ was measured and converted to units per μg *Nb*PR3 protein estimated by Coomassie staining. The lysozyme of chicken egg white (LCEW) was included as a positive control.

An independent experiment confirmed differential activities for 90 of the detected hydrolases (Figs 3, S2; Table S6). We chose five hydrolases with a predicted SP (Almagro Armenteros *et al*., 2019) that showed robustly suppressed activities upon bacterial infection (Fig. 3). We selected three SHs (*Nb*SBT1.7, *Nb*SCPL20 and *Nb*SCPL48), one GH (*Nb*PR3) and one PLCP (*Nb*RD21). Because depletion of suppressed hydrolases is less likely to cause disease phenotypes, we tested whether increased expression could overcome the suppression and uncover the roles of these hydrolases in immunity. We therefore took advantage of the recently developed ‘agromonas’ assay (Buscaill *et al*., 2021), which is based on infections of agroinfiltrated tissues with *Pto*DC3000(*ΔhQ*). We cloned and transiently expressed the five selected hydrolases through agroinfiltration and infected the agroinfiltrated leaves 2 d later with *Pto*DC3000(*ΔhQ*). Bacterial growth was determined 3 d later by plating out dilution series of extracts of infected leaves on selective media. Hydrolase overexpression was confirmed for all the tested hydrolases (Fig. S3A), but only transient expression of *NbPR3* also suppressed bacterial growth (Figs 4a, S3B), suggesting a role for *Nb*PR3 in antibacterial immunity. This ‘agromonas’ infection assay was repeated 10 times, and increased immunity to *Pto*DC3000(*ΔhQ*) upon expression of *Nb*PR3 was found in each of these experiments (Fig. S4; *P* = 8.74 × 10^−9^ over all 10 experiments). Transient expression of *Nb*PR3 also increased resistance to pv *tabaci* 6605 (*Pta*6605), but not against pv *syringae* B728a (*Psy*B728a; Fig. 4a), indicating that *Nb*PR3‐based immunity might be strain specific.

To determine whether antibacterial immunity is dependent on *Nb*PR3 activity, we generated an E92A substitution mutant of *Nb*PR3, replacing the active site Glu by an Ala residue. This *Nb*PR3^E92A^ protein was successfully expressed upon agroinfiltration (Fig. 4b), yet was unable to suppress bacterial growth (Fig. 4c), demonstrating that the intact active site is essential for antibacterial immunity.

*Nb*PR3 belongs to the GH19 hydrolase family and is annotated as an acidic endochitinase. We therefore tested the ability of *Nb*PR3 to degrade a *N*‐acetylglucosamine polymer in an *in vitro* fluorogenic reaction using apoplastic fluid from agroinfiltrated leaves expressing either *Nb*PR3 or its E92A mutant as negative control. A commercial chitinase from the fungus *T. viride* could degrade this substrate, but *Nb*PR3 could not (Fig. 4d), indicating that *Nb*PR3 does not have endochitinase activity.

The cell wall of bacteria contains peptidoglycan, of which the glycan polymer typically consists of alternating residues of β‐(1,4) linked *N*‐acetylglucosamine and *N*‐acetylmuramic acid units. To test whether *Nb*PR3 can hydrolyse peptidoglycan, we monitored the change in the absorbance upon lysis of *Micrococcus lysodeikticus*, which is an established assay for peptidoglycan hydrolysis (Lee & Yang, 2002). However, in contrast to lysozyme from chicken egg white, AF containing *Nb*PR3 could not lyse the bacteria (Fig. 4e), indicating that *Nb*PR3 does not have lysozyme activity.

### Nicotiana‐specific Q112 is required for antibacterial activity

**Fig. 5:**
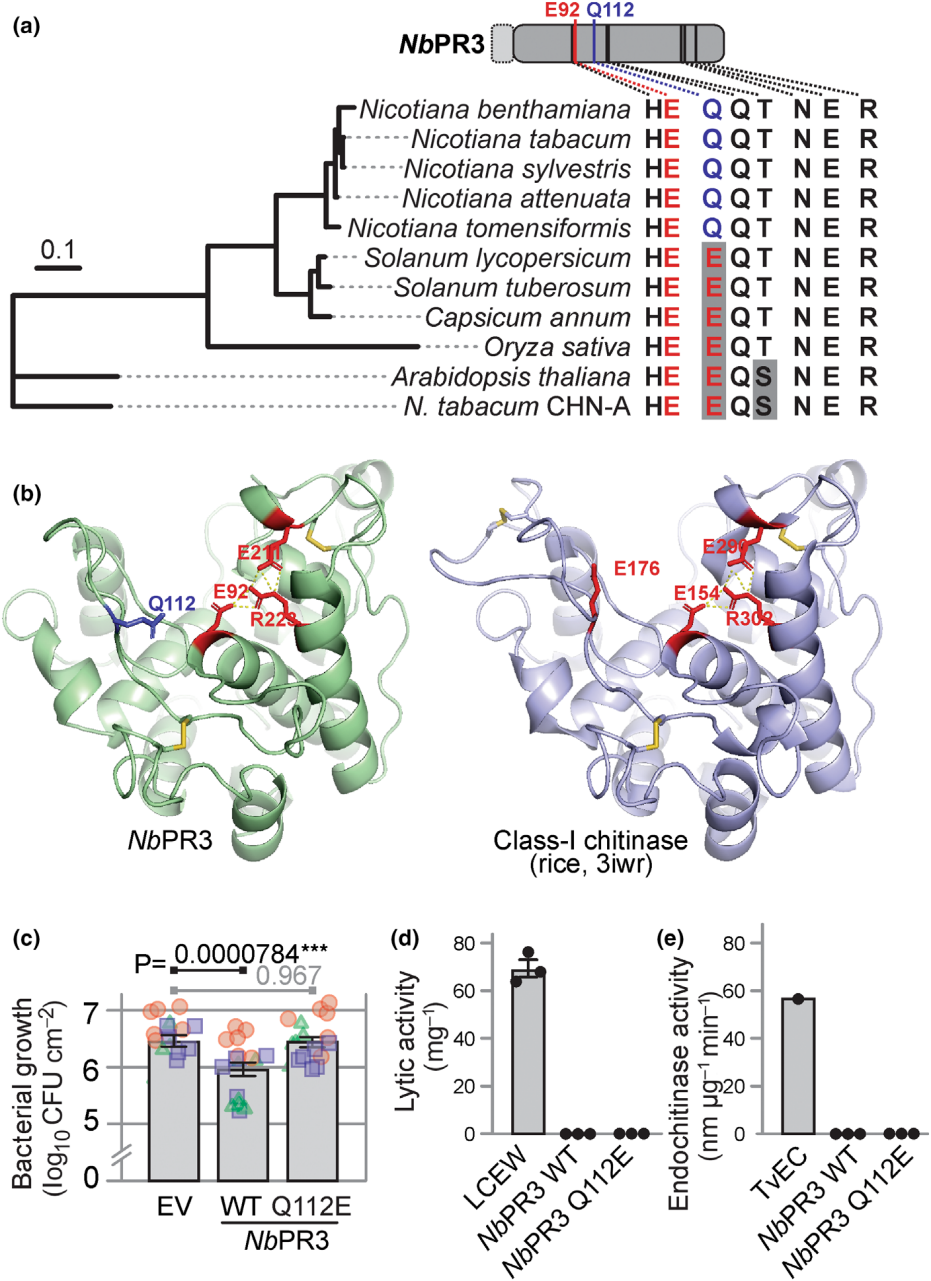
*Nicotiana*‐specific active site Q112 in *Nb*PR3 is essential for antibacterial activity. (a) Conservation of chitinase‐relevant residues in *Nb*PR3 homologs. The second catalytic glutamate of chitinases (red) is replaced by glutamine (blue) in all *Nb*PR3 orthologs of *Nicotiana*. *Nb*PR3 orthologs were identified by Blast searches, aligned by ClustalO (Supporting Information Fig. S6), and used to construct a maximum likelihood phylogenetic tree. Chitinase A (CHN‐A) from *Nicotiana tabacum* was included as the closest related enzyme for which chitinase activity has been demonstrated. The residues relevant for chitinase activity are summarised on the right, showing that the Gln is conserved in *Nicotiana* and is specific for this genus. (b) Model of *Nb*PR3 structure, compared with rice class‐I chitinase. A model of *Nb*PR3 was generated with SWISS Model, using protein data bank 3iwr (Kezuka *et al*., 2010) as a template. Catalytically important residues (red), and the noncanonical Q112 (blue), and three cysteine bridges (yellow) are indicated. (c) The *Nicotiana*‐specific Q112 is essential for antibacterial activity. *Nb*PR3, its Q112E substitution mutant, and the empty vector (EV) control were transiently expressed in *Nicotiana benthamiana* by agroinfiltration, and 2 d later, the same leaf was infiltrated with 10^6^ CFU ml^−1^ *Pto*DC3000(*ΔhQ*). Bacterial population sizes 3 d later are shown in log_10_CFU cm^−2^. Bars show the mean value of 18 biological replicates performed over three separate experiments, and error intervals represent the SE. *P*‐values were calculated by two‐way ANOVA followed by *post hoc* comparison using the Dunnett test to examine the effect of *Nb*PR3 expression on bacterial growth. ***, *P* < 0.001. (d) The Q112E substitution in *Nb*PR3 does not increase lytic activity. Apoplastic fluid (AF) from plants transiently expressing *Nb*PR3 or its Q112E mutant was incubated with *Micrococcus lysodeikticus* cells. The change in A_450_ was measured and converted to units μg^−1^ *Nb*PR3 protein estimated by Coomassie staining. The lysozyme of chicken egg white (LCEW) was included as a positive control. (e) The Q112E substitution in *Nb*PR3 does not increase endochitinase. AF from plants transiently expressing *Nb*PR3 or its Q112E mutant was incubated with 4‐MU‐GlcNac3, and the rate of hydrolysis was measured at 355ex/450em and calculated per μg *Nb*PR3 protein estimated by Coomassie staining. The endochitinase of *Trichoderma viride* (*Tv*EC) was included as a positive control.

We next compared the protein sequence of *Nb*PR3 with chitinase A (CHN‐A) from *Nicotiana tabacum*, the closest related sequence with documented endochitinase activity (Suarez *et al*., 2001). Eight residues have been identified in CHN‐A as being important for endochitinase activity (Garcia‐Casado *et al*., 1998; Tang *et al*., 2004; Chaudet *et al*., 2014; Han *et al*., 2016), and only two of these eight residues are different in *Nb*PR3 (Figs 5a, S5). Notably, whereas the predicted catalytic general acid Glu residue is present in both sequences (*Nb*PR3^E92^ and CHN‐A^E145^), the predicted catalytic general base is absent from *Nb*PR3 (*Nb*PR3^Q112^ vs CHN‐A^E167^). Furthermore, Ser residue S198 in CHN‐A is a Thr residue in *Nb*PR3 (T128), though this substitution is common in the plant kingdom. These sequence polymorphisms indicate that *Nb*PR3 may have a catalytic activity that is different from CHN‐A, but is still likely to bind carbohydrates because the other residues relevant for chitinase activity are conserved. Indeed, structural modelling indicates that *Nb*PR3 may have a similar fold as chitinase‐I (Kezuka *et al*., 2010), but the region carrying Q112 is different from chitinases (Fig. 5b).

Interestingly, Q112 is present in all putative *Nb*PR3 orthologs of the *Nicotiana* genus but is absent in paralogs and in closely related *Solanum* species or Arabidopsis and rice (*Oryza sativa*; Figs 5a, S6), suggesting that this active site substitution occurred in the *Nicotiana* clade. To test the relevance of Q112 for antibacterial activity of *Nb*PR3, we generated the Q112E mutant and tested its ability to suppress the growth of *Pto*DC3000(*ΔhQ*) in the agromonas assay. Importantly, while *Nb*PR3 suppresses bacterial growth, the Q112E substitution abolishes this activity (Fig. 5c), demonstrating that the *Nicotiana*‐specific Q112 is relevant for antibacterial activity. The Q112E mutant of *Nb*PR3 does, however, not gain lytic or endochitinase activity (Fig. 5d), indicating that also other residues contribute to these activities.

#### NbPR3 promotes immunity without inducing PR protein accumulation

**Fig. 6:**
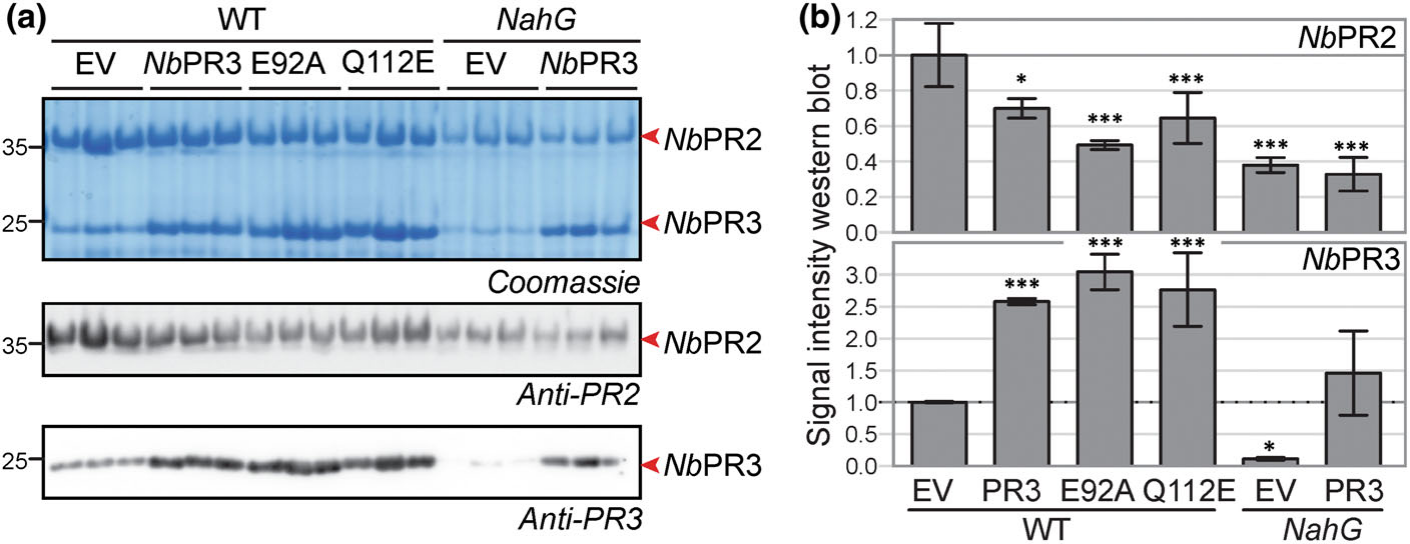
*Nb*PR3 does not promote PR2 protein accumulation. (a) *Nb*PR3 and its mutant derivatives were transiently expressed in WT and *NahG* transgenic *Nicotiana benthamiana* in *n* = 3 independent plants. Apoplastic fluids were isolated 4 d later and analysed by Coomassie staining and western blotting using anti‐PR2 and anti‐PR3 antibodies. (b) Western blot signals were quantified and normalised for the empty vector (EV) control in WT plants. Error bars represent SD of the *n* = 3 replicates. *, *P* < 0.5; ***, *P* < 0.001. *P*‐values are from a one‐way ANOVA with Dunnett correction for multiple comparisons.

Previous work on PR‐Q, the tobacco ortholog of *Nb*PR3, has revealed that transgenic tobacco overexpressing *PR‐Q* also constitutively accumulate PR proteins (Tang *et al*., 2017). To investigate whether *Nb*PR3 expression also induces PR protein accumulation, we monitored the accumulation of PR2, an abundant secreted PR protein in *N. benthamiana*, upon agroinfiltration with empty vector (EV) and *Nb*PR3 and its E92A and Q122E mutant derivatives. The *NahG* transgenic *N. benthamiana* (Wulff *et al*., 2004) was included to determine whether PR protein accumulation was dependent on salicylic acid (SA), which cannot accumulate in *NahG* transgenic plants. Notably, *Nb*PR3 does not induce PR2 levels and the E92A and Q122E mutants of *Nb*PR3 have similar levels of PR2 when compared to EV and *Nb*PR3 (Fig. 6a). In fact, expression of *Nb*PR3 and its mutants even reduces PR2 levels (Fig. 6b), possibly caused by competition on translation and secretion. PR2 levels upon agroinfiltration are much reduced in *NahG* transgenic plants when compared to WT plants and again not increased upon *Nb*PR3 expression (Fig. 6). *Nb*PR3 signals are two–threefold higher in the apoplast of plants transiently expressing *Nb*PR3 and derived mutants (Fig. 6b). Reduced expression of *Nb*PR3 is detected upon agroinfiltration of *NahG* plants, presumably because there is no SA‐regulated expression of endogenous *Nb*PR3, and because the 35S promoter used to express *Nb*PR3 contains a SA‐responsive *as‐1* element (Redman *et al*., 2002). In conclusion, unlike PR‐Q overexpression in tobacco, *Nb*PR3 overexpression by agroinfiltration does not induce PR protein accumulation.

To investigate whether *Nb*PR3 has a direct effect on bacterial growth, we incubated *Pto*DC3000 bacteria in AF isolated from plants expressing EV, *Nb*PR3 and its E92A mutant and monitored bacterial growth over time. Lysozyme mixed with AF of plants agroinfiltrated with EV was included as control. Despite being abundant in AF, *Nb*PR3 did not impact bacterial growth when compared to the EV and E92A controls, whereas lysozyme significantly reduces bacterial growth (Fig. S7), indicating that *Nb*PR3‐derived immunity is not direct.

## Discussion

Most SP‐containing proteins secreted into the apoplast are hydrolytic enzymes. Here, we explored the apoplast of a model plant–pathogen interaction with activity‐based probes and were able to monitor 171 active hydrolases, of which 82 showed increased activity, and 60 had reduced activity upon infection. We have summarised the suppressed hydrolases in Table S7. Disease assays on leaves transiently overexpressing five selected suppressed hydrolases revealed that *Nb*PR3 has antibacterial immunity that requires its active site residue. Despite its annotation as chitinase, *Nb*PR3 has no chitinase or lysozyme activity, presumably because of a E112Q substitution in the active site. This substitution is conserved in *Nicotiana* and is essential for antibacterial activity.

As pathogenic bacteria have adapted to a susceptible host, we anticipate that they will actively suppress secreted hydrolases that may directly or indirectly harm them. Therefore, consistently suppressed hydrolases might be components of the plant immune system that are targeted by pathogen‐derived inhibitors. We identified 60 suppressed hydrolases that may play an active role in immunity. Massive changes in protein activities can be caused by altered pH or ion strength, but the influence of these factors was prevented by buffering the AF. Many of the suppressed hydrolases are Cys proteases, which might be suppressed by increased levels of reactive oxygen species (ROS). However, not all Cys proteases are suppressed, indicating that the suppression is caused by selective inhibition rather than ROS. Indeed, we previously showed that *Pto*DC3000(*ΔhQ*) produces Cip1, which is selectively targeting immune‐related Cys proteases of tomato (Shindo *et al*., 2016). Besides Cys proteases, several glycosidases are also suppressed. This includes BGAL1, which is inhibited by a small molecule produced by *Pto*DC3000(*ΔhQ*) (Buscaill *et al*., 2019). However, given the large proportion of changes, we cannot rule out a general apoplastic regulator (i.e. metabolites, specific ions and post‐translational modifications) that might influence global protein activities.

Secreted hydrolases that are suppressed during infection can be important novel components of the extracellular immune system of plants. To test this hypothesis, we overexpressed the hydrolases to overcome the threshold of suppression during infection. Indeed, we found that transient overexpression of *Nb*PR3 results in increased immunity to *Pto*DC3000(*ΔhQ*), confirming that supressed hydrolases may act in immunity. Likewise, we found that transient overexpression of BGAL1 increases bacterial resistance (Buscaill *et al*., 2021). However, not all suppressed hydrolases reduced bacterial growth upon overexpression in our agromonas assay. This might be because their overexpression does not overcome the suppression mechanism or because they act in immunity without reducing bacterial growth in the agromonas assay.

The mechanism by which *Nb*PR3 mediates antibacterial immunity, however, remains mysterious. Overexpression of PR‐Q in tobacco upregulates multiple defence‐related genes, an observation which was thought to originate from an unfolded protein response (UPR) caused by the presumed accumulation of unfolded proteins in the ER (Tang *et al*., 2017). We, however, found that the E92A and Q112E mutants of *Nb*PR3 accumulate equally well but do not trigger immunity, ruling out the UPR being responsible for the observed resistance. That the catalytically important glutamate E92 is essential for the role of *Nb*PR3 in our agromonas assays, implies that catalysis by *Nb*PR3 is required for immunity. However, despite its annotation as an endochitinase, *Nb*PR3 lacks endochitinase activity, at least in our assays. The absence of bacteriolytic activity indicates that *Nb*PR3 does also not hydrolyse peptidoglycan, unlike Arabidopsis LYS1, a secreted GH18 lysozyme that releases immunogenic fragments from peptidoglycan (Liu *et al*., 2014).

The absence of chitinase and lysozyme activities of *Nb*PR3 is probably at least in part caused by the absence of the second catalytic glutamate (E), which is important for chitinase activity (Tang *et al*., 2004) but is a glutamine (Q112) in *Nb*PR3. Interestingly, Q112 is conserved in all *Nicotiana Nb*PR3 orthologs, including tobacco PR‐Q (Payne *et al*., 1990), but is absent in other species. The closest PR3 family members from other angiosperm plants, including rice and Arabidopsis, all have a catalytic glutamate (E) at this position, which indicates that the E112Q substitution occurred in a *Nicotiana* ancestor within the nightshade family *c*. 50 million years ago and was maintained upon speciation. Importantly, we showed that Q112 is essential for antibacterial activity, which implies that an antifungal chitinase in the *Nicotiana* ancestor was *neo*‐functionalised to gain antibacterial activity. This *neo*‐functionalisation must have involved additional substitutions because we found that the Q112E mutant of *Nb*PR3 is insufficient to restore chitinase activity. The fact that the catalytic E92 is still essential for antibacterial activity and that other residues relevant for chitin binding are still present in *Nb*PR3 and its orthologs indicates that *Nb*PR3 might act on a bacterial glycan.

We discovered that the level of active *Nb*PR3 is consistently downregulated upon infection with virulent *Pto*DC3000(*ΔhQ*) (Fig. 3), even though the level of the *Nb*PR3 protein increases upon infection (Fig. S8), consistent with being a PR protein. This observation indicates that apoplastic *Nb*PR3 is inhibited during infection by a small molecule or protein secreted by *Pto*DC3000(*ΔhQ*). The active suppression of an antibacterial enzyme is similar to the suppression of BGAL1 by a small molecule inhibitor produced by *Pto*DC3000(*ΔhQ*) (Buscaill *et al*., 2019) and indicates that exploring the apoplastic battlefield with activity‐based proteomics to identify these suppressed hydrolases is an exciting new approach to discover novel components of extracellular immunity in plants.

## Competing interests

None declared.

## Author contributions

DJS, AG and RALvdH planned and designed the research; DJS, AG and PB performed experiments; FK and MK performed proteomic analysis; DK, TK, CJS and MK synthesised probes; DJS and RALvdH wrote the manuscript with help of all authors. DJS and AG contributed equally to this work.

## Supporting information


**Fig. S1** Structure of additional activity‐based probes.
**Fig. S2** Replicate activity‐based proteomics experiment.
**Fig. S3** Transient hydrolase expression and agromonas assays.
**Fig. S4** Replicates of infection assays show increased resistance upon *Nb*PR3 expression.
**Fig. S5** Protein sequence alignment of *Nb*PR3 and endochitinase CHN‐A.
**Fig. S6** Protein alignment of putative *Nb*PR3 orthologs from diverse plant species.
**Fig. S7** *Nb*PR3 does not impact bacterial growth *in vitro*.
**Fig. S8** *Nb*PR3 accumulates in the apoplast upon infection.

**Table S1** Used oligonucleotides.
**Table S2** Used plasmids.

**Table S3** In‐solution digest (ACE_0276).
**Table S4** Protein concentrations in apoplastic fluid.

**Table S5** On‐bead digest (ACE_0236).

**Table S6** On‐bead digest (ACE_0136).

**Table S7** Summary of suppressed hydrolases.
Please note: Wiley is not responsible for the content or functionality of any Supporting Information supplied by the authors. Any queries (other than missing material) should be directed to the *New Phytologist* Central Office.

## Data Availability

The proteomics data have been deposited to the ProteomeXchange Consortium via the PRIDE partner repository (www.ebi.ac.uk/pride/, Perez‐Riverol *et al*., 2022) under accessions PXD039897 (in‐solution digest ACE0276) and PXD034869 (on‐bead digests ACE0136 and ACE0236).
