# Supplementary material for: Activity‐based proteomics uncovers suppressed hydrolases and a neo‐functionalised antibacterial enzyme at the plant–pathogen interface
Source: New Phytol. 2023 Mar 29;241(1):394–408. doi: 10.1111/nph.18857 (PMC10952330; doi:10.1111/nph.18857)
Supplement: Supplementary file 1 — Fig. S1 Structure of additional activity‐based probes. Fig. S2 Replicate activity‐based proteomics experiment. Fig. S3 Transient hydrolase expression and agromonas assays. Fig. S4 Replicates of infection assays show increased resistance upon NbPR3 expression. Fig. S5 Protein sequence alignment of NbPR3 and endochitinase CHN‐A. Fig. S6 Protein alignment of putative NbPR3 orthologs from diverse plant species. Fig. S7 NbPR3 does not impact bacterial growth in vitro. Fig. S8 NbPR3 accumulates in the apoplast upon infection. [file NPH-241-394-s006.pdf]

New Phytologist Supporting Information

Title: Activity-based proteomics uncovers suppressed hydrolases and a neo-functionalised antibacterial enzyme at the plant-pathogen interface.

Authors: Daniela J. Sueldo, Alice Godson, Farnusch Kaschani, Daniel Krahn, Till Kessenbrock, Pierre Buscaill, Christopher J. Schofield, Markus Kaiser and Renier A. L. van der Hoorn.

Article acceptance date: 16.2.2023.

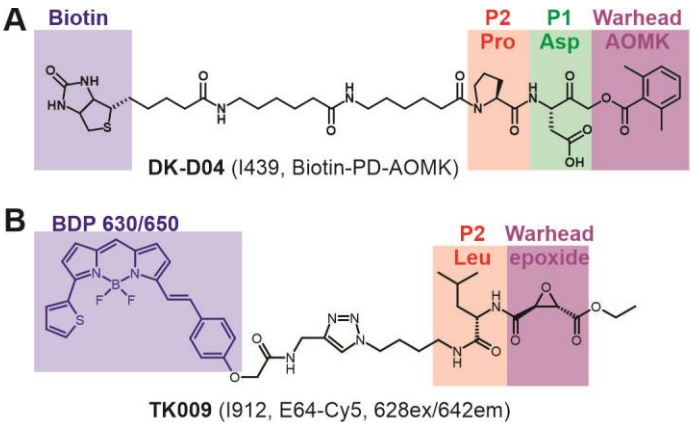

Figure S1 Structure of additional activity-based probes.

(A) Structure of DK-D04 (Biotin-PD-AOMK), a probe for biotinylation of VPEs.

(B) Structure of TK009 (Cy5-E64), a probe for fluorescent labelling of PLCPs.

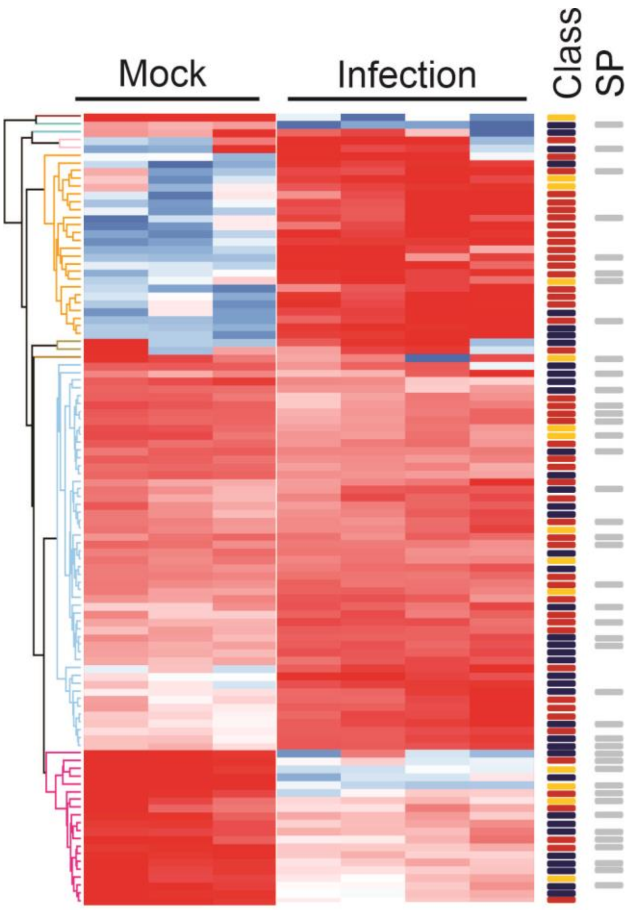

Figure S2. Replicate activity-based proteomics experiment.

Heatmap of 105 active hydrolases detected by activity-based proteomics (experiment ACE136), grouped by category (left) and are annotated as SH(red) / GH(blue) / CP(yellow); hydrolases with a SignalP-predicted signal peptide are marked in grey (right). *N. benthamiana* plants were infected with *Pto*DC3000(*ΔhQ*) or water (mock); apoplastic fluid was collected at 2 days post infection (2dpi). Apoplastic fluid was labelled for active serine hydrolases (SHs), glycosyl hydrolases (GHs) and cysteine proteases (CPs) followed by affinity purification of the labelled proteins and MS analysis.

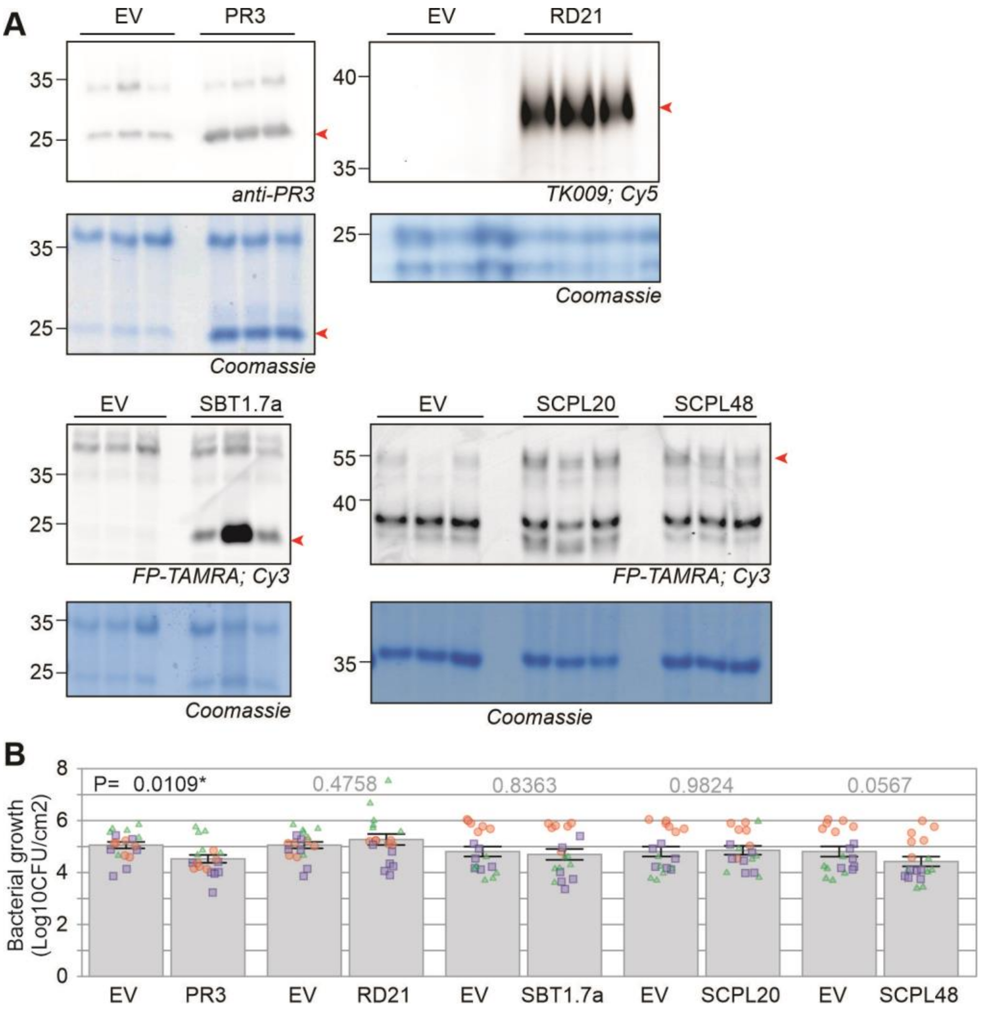

Figure S3 Transient hydrolase expression and agromonas assays.

(A) Confirmation of hydrolase expression. Hydrolase genes were transiently overexpressed by agroinfiltration of *N. benthamiana*. AF was isolated in triplicate at day-4 and labelled with fluorescent probes selective for papain-like proteases (TK009) or Ser hydrolases (FP-TAMRA). Labelled samples were separated on protein gels, which were scanned for fluorescence and stained with Coomassie Blue. *Nb*PR3 accumulation was detected with an anti-PR3 antibody and detected by Coomassie Blue staining. **(B)** *Nb*PR3 reduces bacterial growth. Hydrolases were transiently overexpressed by agroinfiltrations. At day 2, agroinfiltrated leaves were infiltrated with 10^6 CFU/ml *Pto*DC3000(*ΔhQ*). At day 5, leaf extracts were generated, diluted and spotted on plates containing CFC antibiotics to select for *P. syringae*. Bacterial growth is shown in $Log_{10}CFU/cm^2$. Bars show the mean value of 18 biological replicates performed over three separate experiments, and error intervals represent the standard error. P-values were calculated by two-way ANOVA followed by post-hoc comparison using the Dunnett test to examine the effect of hydrolase overexpression on bacterial growth.

A

Experiment	n=	EV-PR3	P value	Fig.
5/2/2020	6	>	0.0038**	4A
19/2/2020	6	>	0.353	4A
17/32020	6	>	0.1004	-
19/8/2020	6	>	0.0389*	4A
25/11/2020	6	>	0.0617	4C
10/2/2021	6	>	0.1004	4C
3/3/2021	6	>	0.4972	4C
13/4/2022	6	>	0.0359*	5C
27/4/2022	6	>	0.05*	5C
11/5/2022	6	>	0.0121*	5C

B

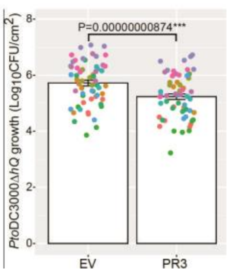

Figure S4 Ten replicate infection assays show increased resistance upon *Nb*PR3 expression. **(A)** Ten experimental replicates, each having n=6 biological replicates. Bacterial growth on *Nb*PR3 expressing leaves was always lower (>) than the EV control, and statistically significant for 5 of the 10 experiments. **(B)** All (6x10=) 60 datapoints of the ten experiments summarised in one figure. The p-value was calculated by a 2-way ANOVA, using the experimental data as a block.

*Nb*PR3 and the empty vector (EV) control were transiently expressed by agroinfiltration. Two days later the same leaf was infiltrated with 10^6 CFU/ml bacteria and bacterial population densities were determined three days later, expressed $Log_{10}CFU/cm^2$.

```
NbPR3   27 IGSIVTSDLFNEMLKNRNDVRCPANGFYTYDAFIAAANSFPGFGTTGDDTARRKEIAAFF 86
CHN-A   80 LGSIISSSMFDQMLKHRNDNACQGKGFYSYNAFINAARSFPGFGTSGDTTARKREIAAFF 139

NbPR3   87 GQTSHETTGGSLSAE--PFTGGYCFVRQNDQ---------------SERYYGRGPIQLTN 129
CHN-A  140 AQTSHETTGGWATAPDGPYAWGYCWLREQGSPGDYCTPSGQWPCAPGRKYFGRGPIQISH 199

NbPR3  130 RNNYEKAGTAIQQDLVNNPDLVATDATISFKTAIWFWMTAQDNKPSSHDVIIGSWTPSAA 189
CHN-A  200 NYNYGPCGRAIGVDLLNNPDLVATDPVISFKSALWFWMTPQSPKPSCHDVIIGRWQPSAG 259

NbPR3  190 DQAANRVPGYGVITNIINGGIECGMGRNDAVEDRIGYYRRYCGMLNVAPGENLDCYNQRN 249
CHN-A  260 DRAANRLPGFGVITNIINGGLECGRGTDSRVQDRIGFYRRYCSILGVSPGDNLDCGNQRS 319

NbPR3  250 FAQG  253
CHN-A  320 FGNG  323
```

Figure S5 Protein sequence alignment of *Nb*PR3 and endochitinase CHN-A.

The mature *Nb*PR3 protein sequence was aligned with the class I endochitinase CHN-A from *N. tabacum*. Residues that differ from *Nb*PR3 (grey), key catalytic residues (red) and other residues relevant for chitinase activity (green) are highlighted.

```
Nbenthamiana       1 -----------------------------------------------MEFSGSPLVLFCC 13
Ntabacum           1 -----------------------------------------------MEFSGSPMALFCC 13
Nattenuata         1 -----------------------------------------------MEFSGSPLALFCC 13
Nsylvestris        1 -----------------------------------------------MEFSGSPMALFCC 13
Ntomentosiformis   1 -----------------------------------------------MEFSGSPLTLFCC 13
Pepper             1 -----------------------------------------------MKFLSSPLAL-TC 12
Potato             1 -----------------------------------------------MEFLGSQMVL-CC 12
Tomato             1 ------------------------------------------------------MVL-CC 5
Arabidopsis        1 MKTNLFLFLIFSLLLSLSSAEQCGRQAGGALCPNGLCCSEFGWCGNTEPYCKQPGCQSQC 60
Rice               1 ------------------------------------------MTTTTTRFVQLAACAAAS 18

Nbenthamiana      14 VFFLFLTGSLAQGIGSIVTSDLFNEMLKNRNDVRCPANGFYTYDAFIAAANSFPGFGTTG 73
Ntabacum          14 VFFLFLTGSLAQGIGSIVTSDLFNEMLKNRNDGRCPANGFYTYDAFIAAANSFPGFGTTG 73
Nattenuata        14 VFFLFLTGSLAQGIGSIVTSDLFNEMLKNRNDGRCPANGFYTYDAFIAAANSFPGFGTTG 73
Nsylvestris       14 VFFLFLTGSLAQGIGSIVTSDLFNEMLKNRNDGRCPANGFYTYDAFIAAANSFPGFGTTG 73
Ntomentosiformis  14 VFFLFLTGSLAQGIGSIVTNDLFNEMLKNRNDGRCPANGFYTYDAFIAAANSFPGFGTSG 73
Pepper            12 VFFLFLTGSLAQDVGSIVTNDLFNNMLKNRDDDRCPAKGFYTYDAFIAAANSFPGFGTTG 71
Potato            12 VFWLFLTGSLAQDVGTIVTSDLFNEMLKNRNDDRCPAKGFYTYDAFIGAANSFSGFGTTG 71
Tomato             6 VFLLFLTGSFAQDVGTIVTSDLFNEMLKNRNDDRCPAKGFYTYDAFIAAANSFPGFGTTG 65
Arabidopsis       61 TPGGTP-PGPTGDLSGIISSSQFDDMLKHRNDAACPARGFYTYNAFITAAKSFPGFGTTG 119
Rice              19 LLAVAASGAAAQGVGSVITQAVFNSMLPNRDNSQCPARGFYTYDAFIAAANSFPAFGTSG 78

Nbenthamiana      74 DDT-ARRKEIAAFFGQTSHETTGGSL-SA-EPFTGGYCFVRQNDQ--------------- 115
Ntabacum          74 DDT-ARRKEIAAFFGQTSHETTGGSL-SA-EPFTGGYCFVRQNDQ--------------- 115
Nattenuata        74 DDT-ARRKEIAAFFGQTSHETTGGSL-SA-EPFTGGYCFVRQNDQ--------------- 115
Nsylvestris       74 DDT-ARRKEIAAFFGQTSHETTGGSL-SA-EPFTGGYCFVRQNDQ--------------- 115
Ntomentosiformis  74 DDT-ARRKEIAAFFGQTSHETTGGSL-SA-EPFTGGYCFVRQNDQ--------------- 115
Pepper            72 DDS-ARKTEIAAFFGQTSHETTGGSL-SADGPFAGGYCFVREGNQ--------------M 115
Potato            72 DDT-ARKKEIAAFFGQTSHETTGGSL-SADGPFAGGYCFLREGNQ--------------V 115
Tomato            66 DDT-ARKKEIAAFFGQTSHETTGGSL-SADGPFAGGYCFVREGNQ--------------M 109
Arabidopsis      120 DTA-TRKKEVAAFFGQTSHETTGGWATAPDGPYSWGYCFKQEQNPASDYCEPSATWPCAS 178
Rice              79 GSAELIRRELAAFFGQTSHETTGGTR-GSSDQFQWGYCFKEEINKA-------------T 124

Nbenthamiana     116 SERYYGRGPIQLTNRNNYEKAGTAIQQDLVNNPDLVATDATISFKTAIWFWMTAQDNKPS 175
Ntabacum         116 SDRYYGRGPIQLTNRNNYEKAGTAIGQELVNNPDLVATDATISFKTAIWFWMTPQDNKPS 175
Nattenuata       116 SDRYYGRGPIQLTNRNNYEKAGTAIGQDLVNNPDLVATDATISFKTAIWFWMTPQDNKPS 175
Nsylvestris      116 SDRYYGRGPIQLTNRNNYEKAGTAIGQELVNNPDLVATDATISFKTAIWFWMTPQDNKPS 175
Ntomentosiformis 116 SDRYYGRGPIQLTNQNNYEKAGNAIRQDLVNNPDLVATDATISFKTAIWFWMTPQDNKPS 175
Pepper           116 GSGFYGRGPIQLTGQSNYDLAGQAIGQDLVNNPDLVATDATVSFKTAIWFWMTPQGNKPS 175
Potato           116 GSGFYGRGPIQLTGQSNYDLAGQAIGQDLVNNPDLVATDATVSFKTAIWFWMTAQGNKPS 175
Tomato           110 GSGFYGRGPIQLTGQSNYDLAGQAIGQDLVNNPDLVATDATVSFKTAIWFWMTAQGNKPS 169
Arabidopsis      179 GKRYYGRGPMQLSWNYNYGLCGRAIGVDLLNNPDLVANDAVIAFKAAIWFWMTAQPPKPS 238
Rice             125 SPPYYGRGPIQLTGQSNYQAAGNALGLDLVGNPDLVSTDAVVSFKTAIWFWMTAQGNKPS 184

Nbenthamiana     176 SHDVIIGSWTPSAADQAANRVPGYGVITNIINGGIECGMGRNDAVEDRIGYYRRYCGMLN 235
Ntabacum         176 SHDVIIGRWTPSAADQAANRVPGYGVITNIINGGIECGIGRNDAVEDRIGYYRRYCGMLN 235
Nattenuata       176 SHDVIIGRWTPSAADQSANRAPGYGVITNIINGGIECGIGRNDAVDDRIGYYRRYCGMLN 235
Nsylvestris      176 SHDVIIGRWTPSAADQAANRVPGYGVITNIINGGIECGIGRNDAVEDRIGYYRRYCGMLN 235
Ntomentosiformis 176 SHDVIIGSWTPSAADQSANRAPGYGVITNIINGGIECGVGPNAAVEDRIGYYRRYCGMLN 235
Pepper           176 CHDVITGQWTPSAADTSANRQPGYGVITNIINGGIECGKGQNPQVEDRIGFYQRYCTILN 235
Potato           176 CHDVITGQWTPSAADTSANRQPGYGVITNIINGGIECGKGQNPQVEDRIGFYRRYCTILN 235
Tomato           170 CHDVITGQWTPSAADASANRQPGYGVITNIINGGIECGKGQNPQVEDRIGFYRRYCTILN 229
Arabidopsis      239 CHAVIAGQWQPSDADRAAGRLPGYGVITNIINGGLECGRGQDGRVADRIGFYQRYCNIFG 298
Rice             185 CHDVILGRWTPSAADTAAGRVPGYGVITNIINGGIECGVGQNDANVDRIGYYKRYCDMLG 244

Nbenthamiana     236 VAPGENLDCYNQRNFAQG------ 253
Ntabacum         236 VAPGENLDCYNQRNFGQG------ 253
Nattenuata       236 VAPGENLDCYNQRNFGQG------ 253
Nsylvestris      236 VAPGENLDCYNQRNFGQG------ 253
Ntomentosiformis 236 VAPGDNLDCYNQRNFAQG------ 253
Pepper           236 VAPGDNLDCYNQKNFAEA------ 253
Potato           236 VAPGDNLDCYTQRNFAEA------ 253
Tomato           230 VAPGDNLDCYDQRNFAEA------ 247
Arabidopsis      299 VNPGGNLDCYNQRSFVNGLLEAAI 322
Rice             245 AGYGSNLDCYNQRNFAS------- 261
```

Figure S6 Protein alignment of putative *Nb*PR3 orthologs from diverse plant species.

ClustalO alignment of putative *Nb*PR3 orthologs from several *Nicotiana* species, pepper, potato, tomato, *Arabidopsis thaliana* and rice. Differences with *Nb*PR3 (grey), key catalytic residues (red) and other residues relevant for chitinase activity (green) are highlighted.

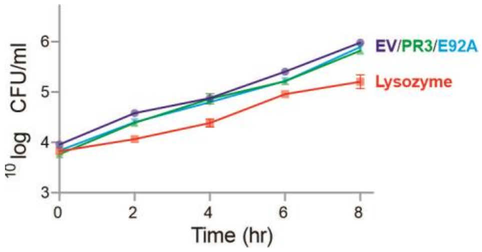

Figure S7 *Nb*PR3 does not impact bacterial growth *in vitro.*

AF from agroinfiltrated plants expressing *Nb*PR3 or its E92A mutant or the empty vector (EV) control, with or without added lysozyme (1 mg/ml), was incubated with OD = 0.001 *Pto*DC3000. Samples were taken at various time points and colony forming units (CFU) was determined by plating out dilution series. Error bars represent standard error of n=3 replicates.

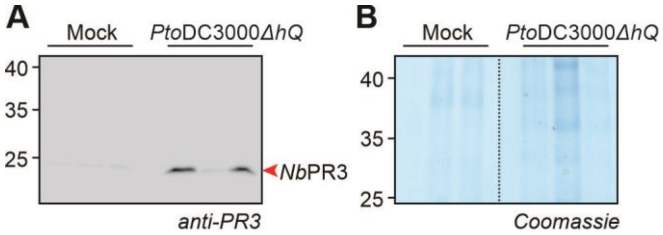

Figure S8 *Nb*PR3 accumulates in the apoplast upon infection.

N. benthamiana leaves were infiltrated with water (mock) or *Pto*DC3000(*ΔhQ*) and AF was isolated from three biological replicates at 2dpi and equal volumes were analysed by western blot with the anti-PR3 antibody **(A)** and on gel, stained with Coomassie Blue **(B)**.
