# Supplementary material for: Activity‐based proteomics uncovers suppressed hydrolases and a neo‐functionalised antibacterial enzyme at the plant–pathogen interface
Source: New Phytol. 2023 Mar 29;241(1):394–408. doi: 10.1111/nph.18857 (PMC10952330; doi:10.1111/nph.18857)
Supplement: Supplementary file 2 — Table S1 Used oligonucleotides. Table S2 Used plasmids. [file NPH-241-394-s005.pdf]

New Phytologist Supporting Information

Title: Activity-based proteomics uncovers suppressed hydrolases and a neo-functionalised antibacterial enzyme at the plant-pathogen interface.

Authors*:* Daniela J. Sueldo, Alice Godson, Farnusch Kaschani, Daniel Krahn, Till Kessenbrock, Pierre Buscaill, Christopher J. Schofield, Markus Kaiser and Renier A. L. van der Hoorn.

Article acceptance date: 16.2.2023

Table S1 Used oligonucleotides.

**Name**	**Purpose**	**Sequence (5´ to 3´)**
PR3_F	Clone *Nb*PR3 from cDNA	tggtggtctcaaATGGAGTTTTCTGGATCACCACTGG
PR3_R	Clone *Nb*PR3 from cDNA	ttcgtggtctcaaagcCTAGCCCTGGGCGAAGTTCCTT
RD21_F	Clone *Nb*RD21 from cDNA	tggtggtctcaaATGGCAAATCATAGCTCCACTCTCA
RD21_R	Clone *Nb*RD21 from cDNA	ttcgtggtctcaaagcTCAAGAACTGCTCTTCCGTCCT
SBT1.7a_F1	Clone *Nb*SBT1.7a from cDNA, remove BsaI	tggtggtctcaaATGTCTAGATACCCAGTAATACTTG
SBT1.7a_R1	Clone *Nb*SBT1.7a from cDNA, remove BsaI	ttcgtggtctcaACCCTTCTCCATAGCAGCAA
SBT1.7a_F2	Clone *Nb*SBT1.7a from cDNA, remove BsaI	tggtggtctcaGGGTATTTTGGTGTCTTGTTCAGC
SBT1.7a_R2	Clone *Nb*SBT1.7a from cDNA, remove BsaI	ttcgtggtctcaaagcTCATGTCCAAGAAATTGCCACAG
SCPL20_F	Clone *Nb*SCPL20 from cDNA	tggtggtctcaaATGGCAAGAATAAATGC
SCPL20_R	Clone *Nb*SCPL20 from cDNA	ttcgtggtctcaaagcTCATATGTTCTTGCCCTC
SCPL48_F	Clone *Nb*SCPL48 from cDNA	tggtggtctcaaATGGCTTCTTCTTTCTCTC
SCPL48_R	Clone *Nb*SCPL48 from cDNA	ttcgtggtctcaaagcCTAGGACAATTTGCCTTG
E92A_F	Mutate E92A in *Nb*PR3 by SDM	cttctcatgcaactactggtggttccctgagtgcagaacc
E92A_R	Mutate E92A in *Nb*PR3 by SDM	ccaccagtagttgcatgagaagtttgaccgaaaaaggcagc
Q112E_F	Mutate Q112E in *Nb*PR3 by SDM	gctttgttcgggaaaatgaccagagtgaaagatattatggtagaggacccatcc
Q112E_R	Mutate Q112E in *Nb*PR3 by SDM	cactctggtcattttcccgaacaaagcaatatcctcctgtaaatggttctgc

Table S2 Used plasmids.

**Name**	**Description**	**Reference**
pICH51288	pL0M-PU-35S-TMV-3-5128835S	Engler *et al.*, 2014
pICH41414	pL0M-T-35S-1-4141435S	Engler *et al.*, 2014
P19	Binary 35S::P19	Van der Hoorn *et al.*, 2003
pJK001c	Binary empty vector (EV)	Paulus *et al.*, 2020
pAG001	Binary 35S::*Nb*PR3	This work
pPB034	Binary 35S::*Nb*RD21	This work
pAG003	Binary 35S::*Nb*SBT1.7a	This work
pAG004	Binary 35S::*Nb*SCPL20	This work
pAG005	Binary 35S::*Nb*SCPL48	This work
pAG006	Binary 35S::*Nb*PR3^{E92A}	This work
pAG007	Binary 35S::*Nb*PR3^{Q112E}	This work

Table S4 protein concentrations in AF.

Protein concentration (mg/mL)		Ratio
**Mock**	**ΔhQ**	**(ΔhQ/WT)**
0.136	0.864	6.35
0.078	0.921	11.8
0.042	0.783	18.6
0.058	1.148	19.8
